# *Parametarhizium* (*Clavicipitaceae*) gen. nov. With Two New Species as a Potential Biocontrol Agent Isolated From Forest Litters in Northeast China

**DOI:** 10.3389/fmicb.2021.627744

**Published:** 2021-02-19

**Authors:** Siyu Gao, Wei Meng, Lixiang Zhang, Qun Yue, Xu Zheng, Lijian Xu

**Affiliations:** ^1^College of Advanced Agriculture and Ecological Environment, Heilongjiang University, Harbin, China; ^2^College of Life Science, Northeast Forestry University, Harbin, China; ^3^Biotechnology Research Institute, Chinese Academy of Agricultural Sciences, Beijing, China

**Keywords:** 3 new taxa, taxonomy, phylogeny, biopesticide, *metarhizium*, entomopathogenic fungi

## Abstract

A novel genus *Parametarhizium* with two new entomopathogenic species, *Parametarhizium changbaiense* and *Parametarhizium hingganense*, was introduced based on their morphological characteristics and a multigene phylogenetic analysis, which were isolated from the forest litters collected in Northeast China. To infer their phylogenetic relationships, a six-gene dataset consisting of DNA fragments of [nuclear small subunit rDNA (SSU) + LSU + TUB + TEF + RPB1 + RPB2] was used for phylogenetic analysis, including 105 related fungi. The new genus *Parametarhizium* formed a monophyletic clade basal to *Metarhizium* and its related genera (formerly *Metarhizium sensu lato*). *Parametarhizium* can be morphologically distinguished from related genera by the combination of the following characteristics: formation of white to yellow colonies on different media, candelabrum-like arrangement of cylindrical or obpyriform phialides, and small subglobose to ellipsoidal conidia. Both *P. hingganense* and *P. changbaiense* exhibited anti-insect activities against three farmland pests *Monolepta hieroglyphica*, *Callosobruchus chinensis*, and *Rhopalosiphum maidis*. This is the first report of entomopathogenic fungi exhibiting the anti-insect activity against *Mo. hieroglyphica*.

## Introduction

Fungi are diverse, with over 144,000 species (Willis, 2018), and about 2,000 new species are described every year (Hawksworth and Lücking, 2017). Fungi are a valuable natural resource and can be applied in agriculture and industry, such as biocontrol agents and antibiotic production (Aly et al., 2011; Filizola et al., 2019; Hyde et al., 2019). They can be used to control farmland pests as a promising green alternative for chemical pesticides (Tulloch, 1976; Zimmermann, 2007; Brunner-Mendoza et al., 2018). However, only a few fungal genera are commercially developed as biocontrol agents such as *Metarhizium*, *Beauveria*, and *Trichoderma* (Zimmermann, 1993). Besides the species from the genera *Metarhizium*, *Beauveria*, and *Trichoderma*, the anti-insect activities of other fungi also deserve to be investigated extensively, especially newly discovered fungal species.

The family Clavicipitaceae (Ascomycota, Hypocreales) is a large group of fungi with 48 genera (including six new genera erected in 2020) and over 500 species^1^ (Mongkolsamrit et al., 2020; Wijayawardene et al., 2020). Clavicipitaceae is morphologically characterized by cylindrical asci, thickened ascus apices, and filiform and multiseptate ascospores that tend to often disarticulate at maturity (Rogerson, 1970; Sung et al., 2007a). Fungi of Clavicipitaceae can grow in plants or invertebrates as symbionts or parasites, such as *Epichloë* spp. (symbionts of grasses), ergot fungi (*Claviceps* spp.) that parasitize ears of cereals, and *Metarhizium* spp. (parasites of insects) (Sung et al., 2007b; Schardl et al., 2013; Mongkolsamrit et al., 2020). *Metarhizium* is a ubiquitous genus of entomopathogenic fungi, being first discovered and erected in 1879 (Metchnikoff, 1879). Over the next 100 years, various new *Metarhizium* species were described based on their morphology characterized by dark spores and branched conidiophore with whorls of two to four phialides. Some *Metarhizium* species had been developed and become famous commercial biocontrol agents, such as *Metarhizium anisopliae*, *Metarhizium brunneum*, and *Metarhizium robertsii* (Meyling and Eilenberg, 2007; Zimmermann, 2007; Castrillo et al., 2011). With the increase in the fungal strain discovery of *Metarhizium* and its related genera, it is difficult to identify cryptic species by morphology alone (Bischoff et al., 2009; Luangsa-ard et al., 2017; Mongkolsamrit et al., 2020). Subsequently, multigene phylogenetic studies resulted in the delimitation of various lineages of *Metarhizium* into new species and genera (Bischoff et al., 2006, 2009; Kepler et al., 2014). Until 2014, according to the combination of phylogenetic and morphological studies, *Metarhizium* was expanded to over 30 species, which included members of the *Metarhizium flavoviride* and *M. anisopliae* complex as well as five additional *Metarhizium* spp. forming a more basal lineage within *Metarhizium* (Kepler et al., 2014). Since then, a dozen new species of *Metarhizium* were discovered including *Metarhizium baoshanense* (Chen et al., 2018), *Metarhizium argentinense* (Gutierrez et al., 2019), *Metarhizium purpureogenum* (Nishi et al., 2017), *Metarhizium bibionidarum* (Nishi et al., 2017), *Metarhizium chaiyaphumense* (Luangsa-ard et al., 2017), *Metarhizium kalasinense* (Luangsa-ard et al., 2017), *Metarhizium lepidopterorum* (Chen et al., 2019), *Metarhizium rongjiangense* (Chen et al., 2019), and *Metarhizium dendrolimatilis* (Chen et al., 2017). Recently, a more extensive multigene phylogenetic study of the Clavicipitaceae enabled the resolution of some of the basal lineages of *Metarhizium* (Mongkolsamrit et al., 2020). As a consequence, *Metarhizium* was redefined, leading to the erection of six new related genera, which are well-supported monophyletic groups based on the multigene phylogenetic analysis. Morphologically, the species of *Metarhizium* and related genera can be distinguished by the arrangement and shape of the phialides; the color, shape, and size of the conidia; and other characteristics (Mongkolsamrit et al., 2020). Although *Metarhizium* is one of the best-studied entomopathogenic fungi with some species being marketed as biocontrol agents, most of the newly described species related to *Metarhizium* in the last decade were rarely screened for their anti-insect activities.

Entomopathogenic fungi are mainly isolated from their hosts directly, which, however, are difficult to find in the field. Therefore, we started looking for alternative sources to find fungi with anti-insect activities. Forest litters naturally include fallen leaves, dead insects, feces, and diverse microorganisms such as fungi. Our previous studies showed that forest litter is a very promising substrate for the discovery of new fungal species in general as exemplified by the recently described *Myxotrichum albicans* (Liang et al., 2019). In this study, we identified and described a new genus related to *Metarhizium* represented by two new candelabrum-like species based on morphology and a multilocus phylogenetic reconstruction. In addition, their potential as biocontrol agents was evaluated by applying a spore-based anti-insect assay.

## Materials and Methods

### Sample Collection

Forest litters were collected in the Changbai Mountains, Jilin province, China (42°24’N, 128°06’E), in October 2017 and Greater Hinggan mountains, Heilongjiang province, China (52°20’N, 124°42’E) in September 2018. The components of forest litters were mostly fallen leaves and a few twigs. The Changbai Mountains is a typical temperate-continental climate zone with an over 300-year-old broad-leaved Korean pine mixed forest. The dominant tree species are *Pinus koraiensis* Siebold et Zucc., *Tilia amurensis* Rupr., *Fraxinus mandshurica* Rupr., and *Quercus mongolica* Firsch. ex Turcz. The Greater Hinggan mountains are located within the cold temperate continental climate zone and consist of *Populus davidiana* Dode, *Betula platyphylla* Sukaczev, *Larix gmelinii* Rupr., and *Pinus sylvestris* L. as the dominant tree species.

### Fungal Medium

The fungal medium comprised potato dextrose agar (PDA; 200 g potato, 20 g glucose, 20 g agar, and 1 L distilled water); 1/4 PDA (50 g potato, 5 g glucose, 20 g agar, and 1 L distilled water); Sabouraud dextrose agar with yeast extract (SDAY; 10 g yeast, 40 g glucose, 10 g peptone, 20 g agar, and 1 L distilled water); oatmeal agar (OA; 30 g oatmeal, 20 g agar, and 1 L distilled water); malt extract agar (MEA; 30 g malt, 20 g agar, and 1 L distilled water). Malt extract powder, agar powder, and yeast extract powder were manufactured by Aoboxing Biotech, Beijing, China. Glucose and peptone were manufactured by Kermel, Tianjin, China.

### Fungal Isolation

The forest litters were packed in sterile envelopes and stored at 4°C until used. Fungi were isolated according to Bills et al. (2004) and Liang et al. (2019). Briefly, a litter sample was pulverized and then subjected to a minisieve filtration. The particles in the range of 105–210 μm were transferred to 10 ml of sterile water. The 0.1 ml suspension of the particles was poured on 1/4 PDA and then cultured at 25°C for 2 weeks. During that time, newly emerging fungal colonies were directly transferred to new PDA plates. By this method, about 500 fungal isolates (data not shown) were obtained. The isolates SGSF125 and SGSF355 were suspected to be undescribed species based on their morphological traits and were used in this study. The specimens were deposited in the Herbarium of Microbiology and Phytopathology, Heilongjiang University (HMPHU 1243 and HMPHU 1244), and the living culture (preserved in a metabolically inactive state) was deposited in China General Microbiological Culture Collection Center (CGMCC 19143 and CGMCC 19144).

### DNA Extraction and PCR

Fungal genomic DNA was extracted from 100 mg of fresh mycelia. DNA extraction followed a previously published protocol (Liang et al., 2019). The DNA nuclear small subunit rDNA (SSU), internal transcribed spacer (ITS), nuclear large subunit rDNA (LSU), beta tubulin (TUB), translation elongation factor 1 alpha (TEF), RNA polymerase II largest subunit (RPB1), and RNA polymerase II second largest subunit (RPB2) were amplified by PCR. PCR was conducted in 50 μl volumes consisting of 25 μl 2 × Es Taq Master Mix, 1 μl template, 2 μl forward primer, 2 μl reverse primer, 1 μl DNA template, and 20 μl ddH_2_O. 2 × Es Master Mix was manufactured by CWBIO, Beijing, China. Primer sequence information is shown in Supplementary Table S1. Sequencing was performed using an ABI 3730 DNA analyzer and the BigDye Terminator mix v. 3.1 (Applied Biosystems, Foster City, California, Unites States). The sequences of the new species were submitted to GenBank (accession number is shown in Supplementary Table S2).

### Physiological Assays

Each isolate was cultured in sterile 90 mm Petri dishes containing 20 ml of PDA and incubated at 20, 25, 30, and 35°C in complete darkness, for 2 weeks. The impact of different pH values (4, 5, 6, 7, 8, and 9) on fungal growth was also tested on PDA. The diameters of fungal colonies were measured every 2 days. After 14 days, 5 ml 0.05% Tween 80 was added to the PDA plates, and conidia were harvested with a sterile cotton swab and deposited into a sterile 10 ml-volume centrifuge tube. The conidial concentration was estimated using a hemocytometer.

### Phylogenetic Analyses

A consensus sequence was obtained by aligning sequences from forward and reverse primers with MEGA v. 7.0 (Kumar et al., 2016) and then queried in the National Center for Biotechnology Information (NCBI) using the BLAST program^2^. The sequences from 105 fungi classified in *Metarhizium* and related genera in the Clavicipitaceae (Kepler et al., 2014; Mongkolsamrit et al., 2020) were obtained from GenBank (Supplementary Table S2) and aligned with MEGA v. 7.0 (ClustalW). Alignments were manually adjusted to allow maximum alignments and minimum gaps. The alignments were concatenated as a dataset for a multigene phylogenetic analysis (Kepler et al., 2014; Mongkolsamrit et al., 2020). The multigene phylogenetic analysis of the aligned sequences used RAxML as an optimality criterion, and statistical support was built on 1,000 bootstrap replicates (Edler et al., 2019). A general time-reversible (GTR) model with a gamma-distributed rate variation was used for the nucleotide partitions in the maximum-likelihood (ML) analysis. Markov chain Monte Carlo (MCMC) was used to estimate posterior probability by MrBayes v. 3.2.4 (Ronquist et al., 2012). Four simultaneous Markov chains were run for 3,000,000 generations (standard deviation of split frequencies less than 0.01), and trees were sampled every 1,000 generations. The first 2,000 trees representing the burn-in phase of the analysis were discarded, and the remaining trees were used to determine posterior probabilities in the majority rule consensus tree.

### Collection of Insects

Adult insects of *Mo. hieroglyphica*, *Rhopalosiphum maidis*, and *Callosobruchus chinensis* were collected from the experimental fields of the Qiqihar Branch of Heilongjiang Academy of Agricultural Sciences from July and August in 2019. The insect adults were collected from fields, placed in a perforated plastic container, and then maintained at 25°C until use.

### Anti-insect Assay

For pathogenicity assays, conidial suspensions were produced using the same method as in the physiological assays. The conidial concentration was adjusted to 1 × 10^8^ conidia/ml using a hemocytometer. Adults of *C. chinensis* were immersed in conidial suspension for 5 s, and 20 adults were inoculated in each treatment (Noble et al., 2018). Adults of *Mo. hieroglyphica* were immersed in conidial suspension for 5 s, and 35 adults were inoculated in each treatment. The effect of fungal isolates on the mortality of *R. maidis* was evaluated by spraying (Hussain et al., 2015; Wu et al., 2020) 1 ml of each conidial suspension on 40 wingless adults per treatment. Tween 80 0.05% was applied as a control treatment. The treated insects were kept in 250 ml flasks with foods and maintained at 25 ± 1°C, 70 ± 5% RH in darkness for 10 days, and the survival of the insects was recorded daily. Cadavers were placed on wet filter paper disks in sterile 90 mm Petri dishes sealed with Parafilm and maintained in an incubator chamber at 25 ± 1°C and 70 ± 5% relative humidity (RH) to investigate mycosis. All the tests were performed in triplicate.

### Statistical Analyses

All data were analyzed using SPSS for Windows Version 25.0 (SPSS Inc., Chicago, IL, United States). Data were shown as the mean ± standard error (SE) from three biological replicates. For multiple comparisons, Tukey’s multiple comparison test was used for significance analysis, and *p*-values equal to 0.05 were considered significant. The significant differences were shown as different letters (such as a, b, and c) in tables and figures.

## Results

### Colony Growth and Conidial Production at Different pH Values and Temperatures

**FIGURE 1:**
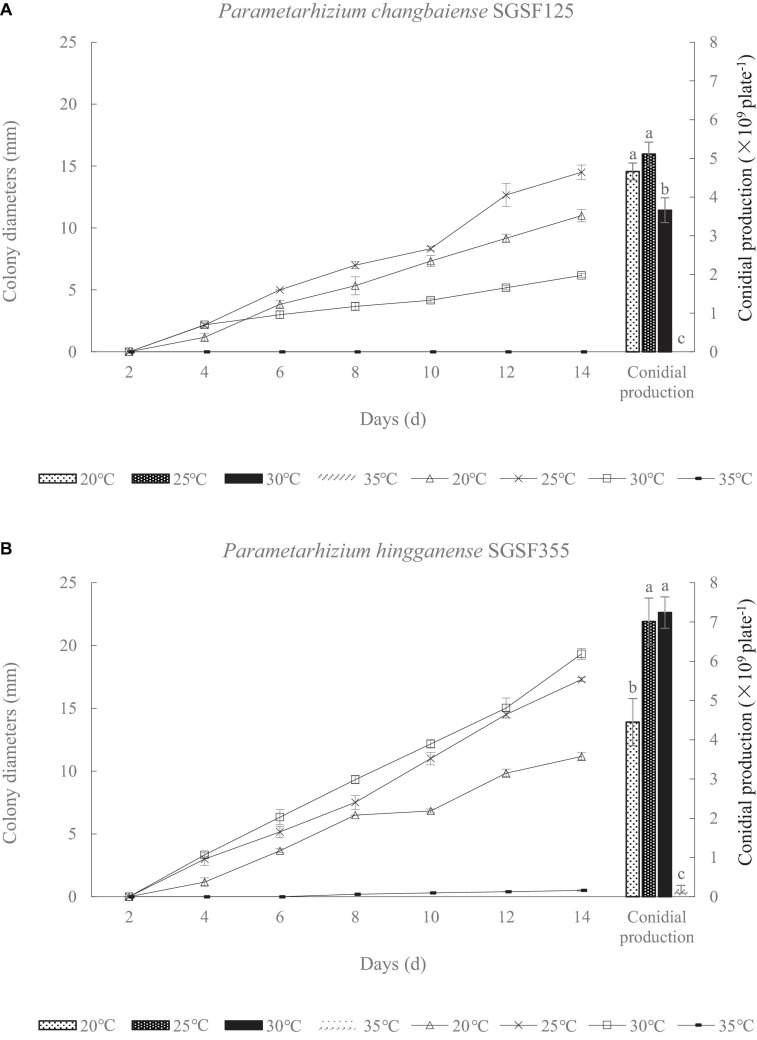
Colony diameter and conidial production of *Parametarhizium chanbaiense* SGSF125 **(A)** and *Parametarhizium hingganense* SGSF355 **(B)** on potato dextrose agar (PDA). Different letters (a, b, and c) above bars indicate significant differences at the 0.05 level.

There are two isolates, SGSF125 and SGSF355, isolated from the forest litters that are suspected as new species related to *Metarhizium* spp. according to their morphological traits. As suspected new species, the optimal pH and temperature of SGSF125 and SGSF355 were tested. The colony diameters and conidial productions of SGSF125 and SGSF355 on PDA at different temperatures, respectively, are shown in Figure 1. The optimal growth temperature of SGSF125 and SGSF355 was 25 and 30°C, respectively. At 25 and 30°C, SGSF355 had a higher growth rate and conidial production than SGSF125. SGSF355 had a wider optimal temperature range than SGSF125, and the conidial production of SGSF355 was similar at 25 and 30°C with no significant difference. The colony diameters of SGSF125 and SGSF355 were 13–18 mm on PDA on the 14th day.

Meanwhile, colony diameters and conidial productions of the two isolates were measured at different pH values within a period of 2 weeks (Table 1). The optimal pH values of SGSF125 and SGSF355 were 5 (Table 1). At the same pH, the colony diameter and conidial production of SGSF355 were higher than SGSF125, except at pH 4 (Table 1).

**TABLE 1:** Colony diameters and conidial productions of *Parametarhizium changbaiense* (SGSF125) and *Parametarhizium hingganense* (SGSF355).

Different pH treatment	Colony diameters		Conidial productions (per plate)	
	SGSF125	SGSF355	SGSF125	SGSF355
pH4	7.2 ± 0.17g	5.2 ± 0.02h	3.23 × 10^9^f	2.69 × 10^9^g
pH5	15.5 ± 0.03b	17.67 ± 0.02a	6.37 × 10^9^c	7.82 × 10^9^a
pH6	14.0 ± 0.03bc	16.17 ± 0.03a	6.34 × 10^9^c	7.36 × 10^9^b
pH7	13.2 ± 0.03cde	14.7 ± 0.02cdef	6.29 × 10^9^cd	6.38 × 10^9^c
pH8	11.7 ± 0.02def	13.3 ± 0.02cd	4.59 × 10^9^e	5.97 × 10^9^d
pH9	11.5 ± 0.00ef	11.3 ± 0.02f	4.56 × 10^9^e	6.11 × 10^9^cd

*Different letters in colony diameters column (a, b, c, d, e, and f) or conidial productions column (a, b, c, d, e, f, and g) indicate significant differences at the 0.05 level.*

### Phylogenetic Analyses

**FIGURE 2:**
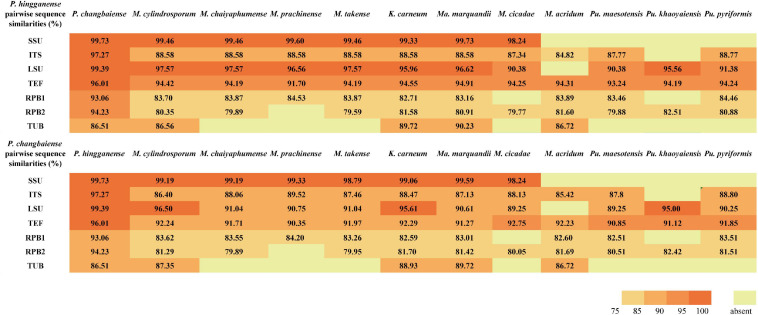
Pairwise nucleotide sequence similarities (in %) for 7-locus from *Parametarhizium* spp. and morphologically similar species.

**FIGURE 3:**
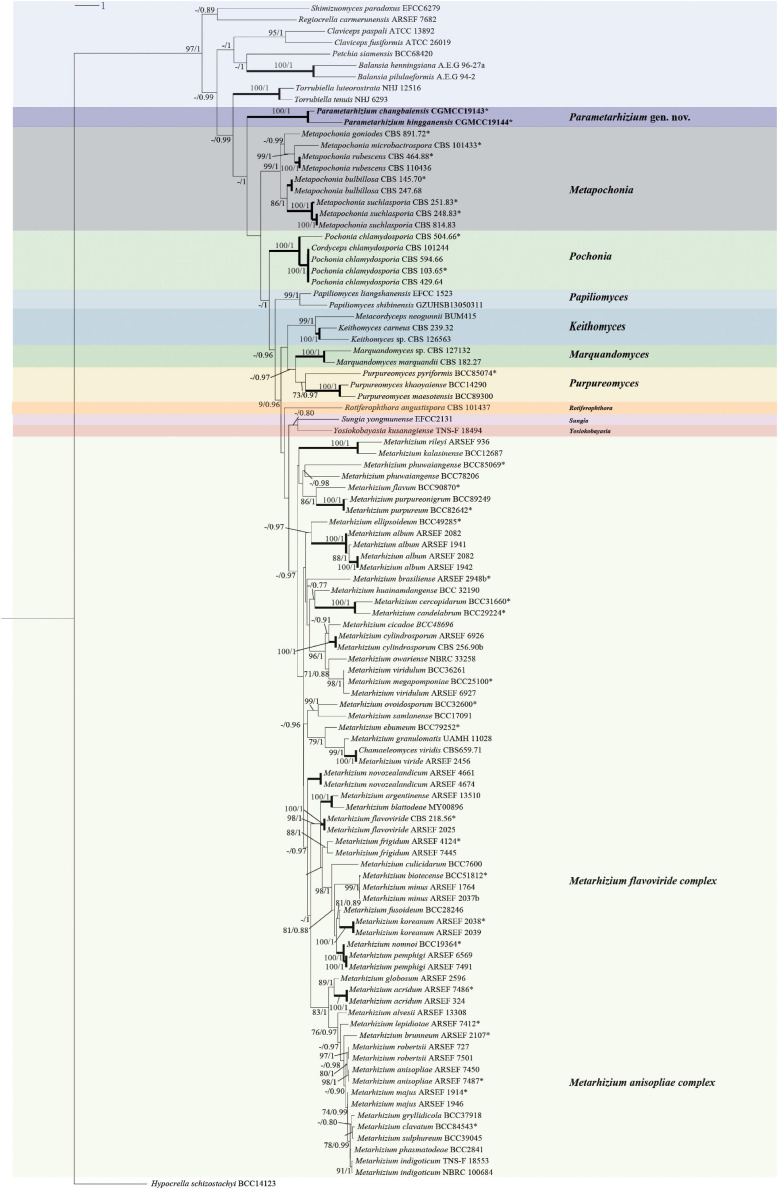
Multigene phylogenetic analysis of *Parametarhizium* and related genera (*Clavicipitaceae*). Phylogenetic based on concatenated Nuclear small subunit rDNA (SSU), Nuclear large subunit rDNA (LSU), Beta tubulin (TUB), Translation elongation factor 1 alpha (TEF), RNA polymerase II largest subunit (RPB1), and RNA polymerase II second largest subunit (RPB2) using maximum likelihood as optimality criterion. ML bootstrap values/Bayesian posterior probability above 70% (MLBS)/0.7 (BPP) are shown on the nodes. *Ex-type material. Branches with 100% (MLBS) and 1.0 (BPP) support are highlighted by thickened lines.

The sequence similarity of seven molecular taxonomic markers SSU, ITS, LSU, TUB, TEF, RPB1, and RPB2 between the two new species was in the range of 93.0–99.7% (except for TUB, 86.5%), indicating their close relationship. In contrast, the similarity to *Metarhizium* species and taxa from other related genera was much lower in particular for the ITS (<89%), RPB sequences (<85%), and TUB (<81%) (Figure 2 and Supplementary Tables S3, S4). All available sequences of the molecular taxonomic markers from the two new and closely related species (*Metarhizium* and the genera related to *Metarhizium*) were combined for a multigene phylogenetic analysis (Supplementary Table S2). The combined dataset concatenated a six-gene dataset consisting of DNA fragments of the SSU (735 bp), LSU (627 bp), TUB (253 bp), TEF (766 bp), RPB1 (492 bp), and RPB2 (778 bp). Sequences of the genus *Hypocrella* were used as an outgroup. ITS sequences were generated only for barcoding purposes and not included in the multigene analyses (Kepler et al., 2014; Mongkolsamrit et al., 2020). Modeltest 3.7 resulted in the selection of the GTR model with a proportion of invariable sites (I) and the gamma distribution shape parameter (G). This model was used in MrBayes 3.2.4 and RAxML GUI 2.0.0–beta.10. Bayesian analyses resulted in 2,000 “burn-in” trees. For the ML analysis in RAxML, the GTRGAMMA model was used for the nucleotide partitions. The phylogenetic analysis (Figure 3) based on a combined dataset comprising SSU, LSU, TUB, TEF, RPB1, and RPB2 showed that the new species formed a monophyletic clade whose bootstrap values (BSs) and Bayesian posterior probability (BPP) were 100% and 1, respectively. This monophyletic was basal to the clade (BS and BPP were 67% and 1, respectively) of *Metapochonia*, *Pochonia*, *Metarhizium*, and other related genera. To include as many genera in Clavicipitaceae as possible, a phylogenetic analysis only based on the LSU sequences from 34 genera in Clavicipitaceae was also carried out (Supplementary Figure S1). The new species also formed a monophyletic clade in the phylogenetic tree based on the LSU sequences. The isolated position of the new fungal isolates SGSF125 and SGSF355 in the multigene phylogenetic reconstruction together with their unique combination of morphological characteristics prompted us to erect a novel genus named *Parametarhizium*.

## Taxonomy

### *Parametarhizium* S. Gao, W. Meng, Li Xiang Zhang, Q. Yue, L. J. Xu, gen. nov.

MycoBank no.: MB 837521

Etymology: based on its close morphological relationship to *Metarhizium.*

Type species: *Parametarhizium hingganense* S. Gao, W. Meng, Li Xiang Zhang, Q. Yue, L. J. Xu, sp. nov.

Diagnosis: Colonies white to yellow, velvety, exudate lemon yellow, sometimes radially sulcate, reverse pale yellow to brown. Conidiophores hyaline, arising from branches of aerial hyphae, with candelabrum-like arrangement of phialides. Phialides, 2–4 in whorls, cylindrical to obpyriform. Conidia subglobose to ellipsoidal, hyaline (1.1–) 1.2–2.2 (–2.8) × (1.0–) 1.6–1.7 (–2.6) μm.

**FIGURE 4:**
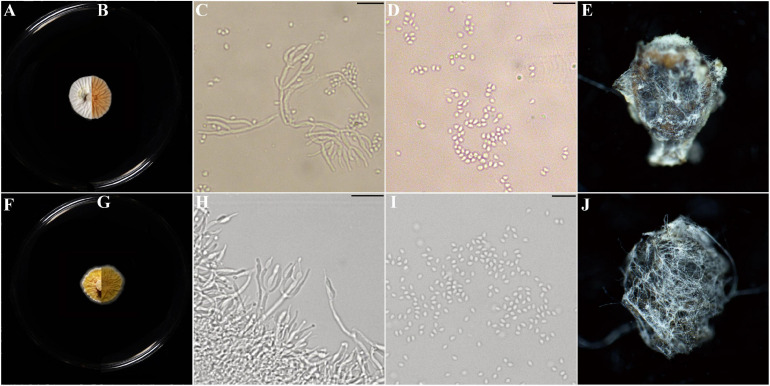
Morphological characters of *Parametarhizium changbaiense* [from ex-holotype China General Microbiological Culture Collection Center (CGMCC) 19143] and *Parametarhizium hingganense* (from ex-holotype CGMCC 19144). **(A,B)** The front and reverse of a *P. changbaiense* colony on PDA after 14 days at 25°C. **(C,D)** The phialides and conidia of *P. changbaiense*. **(E)** *Rhopalosiphum maidis* infected by *P. changbaiense*. **(F,G)** The front and reverse of a *P. hingganense* colony on PDA after 14 days at 25°C. **(H,I)** The phialides and conidia of *P. hingganense*. **(J)** *R. maidis* infected by *P. hingganense.*

Notes: Compared with those of *Metarhizium*, the colonies of *Parametarhizium* are white to yellow (vs. green), and its subglobose-to-ellipsoidal conidia are smaller (<3.3 μm) than most of *Metarhizium* spp. *Parametarhizium* exhibits a candelabrum-like arrangement of phialides, while *Metapochonia* and *Pochonia* have *Verticillium*-like conidiophores, and other related genera show *Nomuraea*-like, *Paecilomyces*-like or *Lecanicillium*-like conidiophores.

Description: Colonies on PDA, SDAY, MEA, OA, white to yellow, never green, radially sulcate, 25°C reaching 13–20 mm in 2 weeks, velvety; exudate lemon yellow; reverse pale yellow to brown. Hyphae hyaline, septate, smooth-walled, 0.8–2.5 μm wide. Conidiophores hyaline, smooth-walled (20–) 34–62 (–100) × (0.9–) 1.5–1.9 (–2.2) μm, arising from branches of aerial hyphae, with whorls of 2–4 phialides. Phialides candelabrum-like arrangement (2.7–) 5.6–17.5 (–26) × (1.2–) 1.4–2.3 (–2.5) μm, cylindrical to obpyriform. Conidia subglobose to ellipsoidal, hyaline to yellow (1.1–) 1.2–2.2 (–2.8) × (1.0–) 1.6–1.7 (–2.6) μm (Figure 4 and Supplementary Figure S2).

### *Parametarhizium changbaiense* S. Gao, W. Meng, Li Xiang Zhang, Q. Yue, L. J. Xu, sp. nov.

MycoBank no.: MB 837522

Etymology: referring to the location where the type material was collected.

Description: Colonies on PDA reaching 13–15 mm in 2 weeks, white, radially sulcate, velvety; reverse brown, radially sulcate. Colonies on SDAY reaching 15–17 mm, pale yellow, wrinkled, velvety, with undulate margin; reverse yellow. Colonies on MEA reaching 15–18 mm, white, flat; reverse brown. Colonies on OA reaching 18–19 mm, flat, initially white, turning lemon yellow due to the production of conidial masses, exudate lemon yellow; reverse pale yellow.

Hyphae hyaline, smooth-walled, 0.8–2.5 μm wide. Conidiophores arising from branches of aerial hyphae, bearing dense whorls of branches, terminating in branches with 2–4 phialides per branch, candelabrum–like arrangement of phialides. Phialides cylindrical (2.7–) 5.6–12.5 (–20) × (1.4–) 1.6–2.2 (–2.5) μm, with a short neck (0.9–) 1.2–5.5 (–7.6) × (0.6–) 0.7–1.1 (–1.3) μm. Conidia unicellular, subglobose to ellipsoidal, hyaline to yellow (1.6–) 2.2 (–2.8) × (1.3–) 1.7 (–2.2) μm (Figure 4 and Supplementary Figure S2).

Type: China, Jilin Province, Changbai Mountains, on litters of forest, October 2017, Li Zeyu and Liu Boyang (holotype HMPHU 1243, culture ex-type CGMCC 19143).

Gene sequences ex-holotype: MN589741 (ITS), MN589994 (LSU), MN590231 (SSU), MN908589 (TEF), MT921830 (TUB), MN917168 (RPB1a), MT921829 (RPB2a).

Sexual morph: not observed

Habitat: forest litters

Distribution: Changbai Mountains, Jilin province, China

Notes: *P. changbaiense* is compared with *P. hingganense*, *Keithomyces aciculare*, *M. flavoviride*, *Marquandomyces marquandii*, *Metarhizium frigidum*, and *Metarhizium suchlasporia*. The conidial size of *P. changbaiense* is smaller than those found in *M. flavoviride*, *M. frigidum*, and *K. aciculare*, and the color of their colonies is not white to yellow. *Ma. marquandii* differs from *P. changbaiense* by the *Paecilomyces*-like conidiophores and purplish colonies, while *M. suchlasporia* exhibits slender phialides. A detailed list of the diagnostic features and sequence similarities in comparison with related species can be found in Table 2 and Figure 2.

**TABLE 2:** Morphological comparison of *Parametarhizium changbaiense* and *Parametarhizium hingganense* with similar species.

Species	Source	Phialides (μ m)	Conidia (μ m)	Colony color	References
*Metarhizium acridum*	Orthoptera	Cylindrical to slightly swollen, 7.0–7.5 × 2.1–2.9	Ovoid, 4.0–5.0 × 2.0–3.2	Dark yellow–green	Bischoff et al., 2009
*Metarhizium cylindrosporum*	Hemiptera (Cicada adult)	5–8 × 3–4.2	Microconidia ovoid, Subglobose, 3.3–6.7 × 3.3–4.2	Grayish–green	Tzean et al., 1993
*Metarhizium chaiyaphumense*	Hemiptera (Cicada nymph)	Clavate, 5–8 × 2–3	Microconidia ovoid, subglobose, 2–4 × 2–3	White–green	Luangsa-ard et al., 2017
*Metarhizium cicadae*	Hemiptera: Cicadidae	Cylindrical, 4–7 × 2–3.5	Microconidia ovoid, ellipsoid, 2–6 × 2.5–4	Dark–green	Mongkolsamrit et al., 2020
*Metarhizium prachinense*	Lepidoptera larva	Ovoid, obpyriform, 3–5 × 2	Subglobose, 3–5 × 1.5–2.5	White–green	Luangsa-ard et al., 2017
*Metarhizium takense*	Hemiptera (Cicada nymph)	Fusiormis, 5.0–8.0 × 2.0–3.0	Subglobose or ovoid, 3.0–5.0 × 2.0–3.0	Green	Luangsa-ard et al., 2017
*Purpureomyces khaoyaiensis*	Lepidoptera larva	*Lecanicillium*–like, 7–18 × 1–3	Ovoid, 2–3 × 1.5–3	White to cream	Mongkolsamrit et al., 2020
*Purpureomyces maesotensis*	Lepidoptera larva	*Lecanicillium*–like, 4–21 × 1.5–2	Ovoid, 3 × 1–2.5	White to lilac	Mongkolsamrit et al., 2020
*Purpureomyces pyriformis*	Lepidoptera larva	*Lecanicillium*–like, 8–22 × 1–2	Ovoid, 2–4 × 1.5–2	White to cream	Mongkolsamrit et al., 2020
*Purpureomyces marquandii*	Soil	Cylindrical, 8–15 × 1.5–2	Ellipsoidal, 2–3 × 1.5–2	Purple	Hywel-Jones, 1994
*Keithomyces carneus*	Soil	Cylindrical, 8–10 × 2.5–3.0	Globose, 3.5–5	White	Frisvad and Samson, 2004
*Parametarhizium changbaiense*	Forest litter	Cylindrical, 5.6–12.5 × 1.6–2.2	Subglobose, ellipsoid, 1.6–2.8 × 1.3–2.2	White to yellow	This study
*Parametarhizium hingganense*	Forest litter	Obpyriform, 7.0–17.5 × 1.4–2.3	Subglobose, ellipsoid, 1.1–3.3 × 1.0–2.6	Yellow	This study

### *Parametarhizium hingganense* S. Gao, W. Meng, Li Xiang Zhang, Q. Yue, L. J. Xu, sp. nov.

MycoBank no.: MB 837523

Etymology: referring to the location where the type material was collected.

Description: Colonies on PDA reaching 17–18 mm in 2 weeks, yellow, radially sulcate, velvety, with undulate margin; exudate lemon yellow; reverse yellow, radially sulcate. Colonies on SDAY reaching 17–20 mm, pale yellow, wrinkled, velvety, with undulate margin; reverse brown, wrinkled. Colonies on MEA reaching 15–18 mm, lemon yellow, radially sulcate, velvety; reverse brown, radially sulcate. Colonies on OA reaching 18–19 mm, flat, initially cream, turning lemon yellow due to the production of conidial masses; reverse pale yellow.

Hyphae hyaline, septate, smooth-walled, 0.8–2.5 μm wide. Conidiophores arising from branches of aerial hyphae, bearing dense whorls of branches, terminating in branches with 2–4 phialides per branch, candelabrum-like arrangement of phialides. Phialides obpyriform (3.5–) 7.0–17.5 (–26) × (1.2–) 1.4–2.3 (–2.5) μm, with a long distinct neck, (1.6–) 2.5–7.1 (–8.5) × (0.5–) 0.6–1.1 (–1.2) μm. Conidia unicellular, subglobose to ellipsoidal, hyaline to yellow, (1.1–) 1.2 (–3.3) × (1.0–) 1.6 (–2.6) μm (Figure 4 and Supplementary Figure S2).

Type: China, Heilongjiang province, Greater Hinggan mountains, on litters of forest, September 2018, Li Zeyu and Liu Boyang (holotype HMPHU 1244, culture ex-type CGMCC 19144).

Gene sequences ex-holotype: MN055703 (ITS), MN061635 (LSU), MN055706 (SSU), MN065770 (TEF), MN061672 (TUB), MN917170 (RPB1a), MT939494 (RPB2a).

Sexual morph: not observed

Habitat: forest litters

Distribution: Greater Hinggan mountains, Heilongjiang province, China.

Notes: *P. hingganense* is compared with *P. changbaiense*, *Keithomyces carneum*, *Ma. marquandii*, *Metarhizium globosum*, *Metarhizium minus*, and *Metarhizium blattodeae*. The phialides arrangement of *K. carneum* and *Ma. marquandii* is *Paecilomyces*-like, but *P. hingganense* possesses candelabrum-like conidiophores. The conidia of *M. blattodeae*, *M. globose*, and *M. minus* are bigger than those in *P. hingganense* and have different shapes. *P. changbaiense* and *P. hingganense* differ mainly in the shape of their phialides and colony characteristics. The phialides of *P. changbaiense* are cylindrical with a short neck, while those of *P. hingganense* are obpyriform with a long distinct neck. Colonies of *P. changbaiense* are white on PDA compared to the yellow colonies of *P. hingganense* (Figure 4). A detailed list of the diagnostic features and sequence similarities in comparison with related species can be found in Table 2 and Figure 2.

### Anti-insect Potentials of *P. changbaiense* and *P. hingganense*

Given the similarity to the core *Metarhizium*, the anti-insect abilities of *P. changbaiense* and *P. hingganense* were tested. *P. changbaiense* and *P. hingganense* were both pathogenic to *Mo. hieroglyphica*, *C. chinensis*, and *R. maidis* under laboratory conditions. The external white mycelia of *P. changbaiense* and *P. hingganense* on the cadavers of three tested insects firstly emerged from the abdomen and then gradually surrounded the insects. In contrast to green cadavers caused by most *Metarhizium* spp. such as *M. anisopliae* and *M. robertsii*, the color of cadavers caused by *P. changbaiense* and *P. hingganense* was white (Figure 4 and Supplementary Figure S3). The mortality of *P. changbaiens*e and *P. hingganense* to above their farmland insects was higher than 85%, and there was no significant difference between *P. changbaiens*e and *P. hingganense* with the exception that *P. changbaiense* against *C. chinensis* was 61.67% (Table 3).

**TABLE 3:** Mortality rates, LT_50_ (median lethal time) and cadaver rate of *Parametarhizium* spp. isolates.

Host species	Strain no.	Mortality (%)	Median lethal time (days)	Cadaver rate (%)
*Monolepta hieroglyphica*	*P. changbaiense*	99.05 ± 0.95a	3.13	95.56 ± 0.2a
	*P. hingganense*	99.05 ± 0.95a	2.92	98.89 ± 0.2a
	Control	32.38 ± 0.95c	11.17	0
*Callosobruchus chinensis*	*P. changbaiense*	61.67 ± 8.81b	9.41	73.33 ± 0.2c
	*P. hingganense*	98.33 ± 1.66a	6.37	85.00 ± 0.3b
	Control	40 ± 2.88c	10.67	0
*Rhopalosiphum maidis*	*P. changbaiense*	100 ± 0.00a	0.85	71.67 ± 0.2c
	*P. hingganense*	100 ± 0.00a	0.54	80.83 ± 0.2b
	Control	38.33 ± 2.2c	4.08	0

*Different letters (a, b, and c) in the same column indicate significant differences at the 0.05 level.*

**FIGURE 5:**
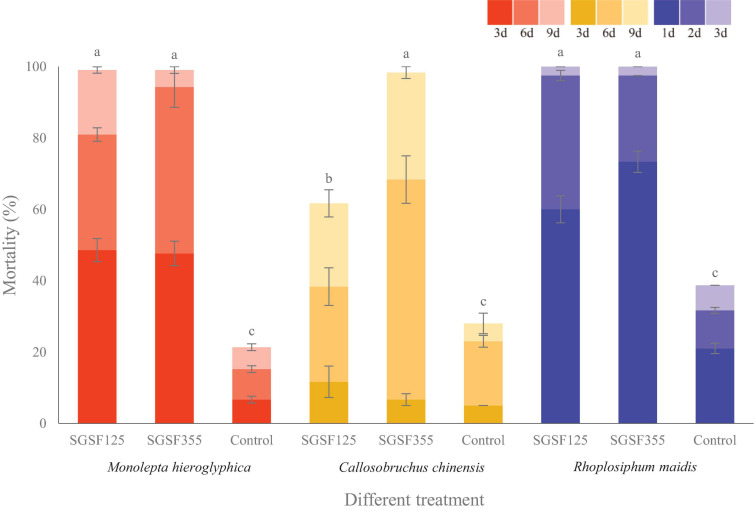
Anti-insect activity screening of *Parametarhizium changbaiense* and *Parametarhizium hingganense* on the different day. Red columns are *Monolepta hieroglyphica*, yellow columns are *Callosobruchus chinensis*, and blue columns are *Rhopalosiphum maidis* Different letters (a, b, and c) above bars indicate significant differences at the 0.05 level.

With respect to the cadaver rate, all of them were higher than 70%. The cadaver rate of *P. hingganense* to *C. chinensis* and *R. maidis* is significantly higher than that of *P. changbaiense* SGSF125. The median lethal time of *P. changbaiense* and *P. hingganense* strongly varied between the different insects but was significantly shorter than that of the control. Most of the *R. maidis* individuals died within a day after the treatment (4 days in the control), while *C. chinensis* individuals survived for around 6 days when treated with *P. hingganense* and 9 days when treated with *P. changbaiense* (11 days in the control). The effect on *Mo. hieroglyphica* was even more remarkable with a median lethal time of 3 days for both treatments compared to 11 days in the control (Figure 5). The results of the anti-insect assays implied that *P. changbaiense* and *P. hingganense* are non-host-specific entomopathogens and thus offer a biocontrol potential against farmland pests.

## Discussion

Although the discovery of new fungal taxa is on the rise, mycotaxonomy is still an ongoing challenge when introducing new fungal species. Jeewon and Hyde (2016) provided many recommendations for the appropriate use of phylogenetic and morphological data in species delineation, and in this study, we followed the guidelines to establish our new taxa. We proposed one new genus with two new species which are related to members of the *Metarhizium* complex (formerly *Metarhizium sensu lato*). According to the pairwise nucleotide sequence similarities for seven loci (Figure 2), the close relatives of *P. changbaiense* and *P. hingganense* are genus *Metarhizium* and its related genera (formerly *Metarhizium sensu lato*). Currently, the phylogenetic relationships of *Metarhizium* and its related genera are inferred based on DNA sequence analyses from multigenes (Mongkolsamrit et al., 2020). Since the related genera are not in the clade of the core *Metarhizium* forming monophyletic clades and their morphological traits are different from core *Metarhizium*, they have been renamed as other new genera, respectively, such as *Papiliomyces* and *Keithomyces* (Mongkolsamrit et al., 2020). The phylogeny shown in Figure 3 was constructed from DNA sequence analyses of the SSU, LSU, TUB, TEF, RPB1, and RPB2 regions, which supported the current taxonomy of *Metarhizium* and its related genera erected in 2020. This phylogenetic analysis also strongly supported that *P. changbaiense* and *P. hingganense* form a monophyletic clade similar with the related genera erected in 2020 (Mongkolsamrit et al., 2020). Combined with morphological results, it is proposed that a new genus, *Parametarhizium*, is erected.

The morphological traits of *P. changbaiense* and *P. hingganense* are compared with their close species, including phialides arrangements, sizes and shapes of conidia, and colony colors (Table 2). The conidia of *Parametarhizium* are subglobose, and their size is smaller than that of *Metarhizium*, and the colony of *Parametarhizium* is yellow or white with the exudate compared with the green colony of *Metarhizium*. With respect to phialides arrangements, *Metarhizium* and its related genera have four kinds, candelabrum-like, *Verticillium*-like, *Paecilomyces*-like, and *Lecanicillium*-like. *Parametarhizium* is candelabrum-like. *Metapochonia* and *Pochonia* are *Verticillium*-like. *Keithomyces* and *Marquandomyces* are *Paecilomyces*-like. *Purpureomyces* and *Papiliomyces* are *Lecanicillium*-like. The conidiophores of *Sungia* erect with solitary, awl-shaped phialides. Furthermore, *Metapochonia*, *Pochonia*, *Sungia*, and *Marquandomyces* produce chlamydospores, but *Parametarhizium* on PDA, SDAY, MEA, and OA could not produce chlamydospores. In addition, the colony color of *Parametarhizium* is white to yellow, different from these close genera.

Since the conidia of *Parametarhizium* spp. are subglobose, the morphological traits of the related species producing subglobose conidia are compared and shown in Table 2. The conidia sizes of *Pu. khaoyaiensis*, *Pu. Maesotensis*, *Ma. marquandii* (Banihashemi, 2012) range within 2–3 × 2–3 μm and they are similar to *Parametarhizium*. However, the phialides arrangements of these four species are *Paecilomyces*-like which is different from *Parametarhizium*.

The anti-insect results implied the potentials of *P. hingganense* and *P. changbaiense* as new biocontrol agents against farmland pests. There are some reports about anti-insect activities against *C. chinensis* and *R. maidis* of the entomopathogenic fungi *B. bassiana*, *M. anisopliae*, and *Isaria fumosorosea* (Sajid et al., 2017; Khan et al., 2018). The mortality of *R. maidis* caused by *P. hingganense* and *P. changbaiense* is similar to *M. anisopliae* and higher than the others. To the best of our knowledge, no related studies about anti-insect activity of entomopathogenic fungi against *Mo. hieroglyphica* are published so far. Therefore, the two entomopathogens, *P. hingganense* and *P. changbaiense*, are the first fungal species reported to show anti-insect activity against *Mo. hieroglyphica.*

## Data Availability Statement

The datasets presented in this study can be found in online repositories. The names of the repository/repositories and accession number(s) can be found in the article/Supplementary Material.

## Author Contributions

SG isolated fungi, built up the phylogenetic tree, investigated anti-insect bioassay, and wrote the manuscript. WM designed the experiments, wrote the manuscript, and provided partial funding. LZ identified and provided insects. QY did the chemical analyses and revised the manuscript. XZ collected the insects. LX designed the experiments, identified the fungal isolates, wrote the manuscript, and provided partial funding. All authors discussed the results.

## Conflict of Interest

The authors declare that the research was conducted in the absence of any commercial or financial relationships that could be construed as a potential conflict of interest.

## Footnotes

1 http://www.outlineoffungi.org

2 http://www.ncbi.nlm.nih.gov/

## Abbreviations

BLAST: Basic Local Alignment Search Tool
BPP: Bayesian posterior probability
ITS: Internal transcribed spacer
LSU: Nuclear large subunit
rDNA: LT_50_ Median lethal time
MEA: Malt extract agar
ML: Maximum likelihood
MLBS: Maximum likelihood bootstrap values
NCBI: National Center for Biotechnology Information
OA: Oatmeal agar
PDA: Potato dextrose agar
RPB1: RNA polymerase II largest subunit
RPB2: RNA polymerase II second largest subunit
SDAY: Sabouraud dextrose agar with yeast extract
SGSF125: *Parametarhizium changbaiense* SGSF125 isolate
SGSF355: *Parametarhizium hingganense* SGSF355 isolate
SSU: Nuclear small subunit rDNA
TEF: Translation elongation factor 1 alpha
TUB: Beta tubulin.

## References

[B1] Aly A. H. Debbab A. Proksch P. (2011). Fifty years of drug discovery from fungi. *Fungal Divers.* 50 3–19. 10.1007/s13225-011-0116-y

[B2] Banihashemi Z. (2012). First report of *Paecilomyces marquandii* from Iran. *Rostaniha* 13 207–210.

[B3] Bills G. F. Christensen M. Powell M. Thorn G. (2004). “Saprobic soil fungi,” in *Biodiversity of Fungi: Inventory and Monitoring Method*, eds Mueller G. M. Bills G. F. Foster M. S. (Burlington, MA: Elsevier), 271–302.

[B4] Bischoff J. Rehner S. Humber R. (2006). *Metarhizium frigidum* sp. nov.: a cryptic species of *M. anisopliae* and a member of the *M. flavoviride* complex. *Mycologia* 98 737–745. 10.1080/15572536.2006.11832645 17256577

[B5] Bischoff J. Rehner S. Humber R. (2009). A multilocus phylogeny of the *Metarhizium anisopliae* lineage. *Mycologia* 101 512–530. 10.3852/07-202 19623931

[B6] Brunner-Mendoza C. Reyes-Montes M. Moonjely S. Bidochka M. Toriello C. (2018). A review on the genus *Metarhizium* as an entomopathogenic microbial biocontrol agent with emphasis on its use and utility in Mexico. *Biocontrol. Sci. Techn.* 29 83–102. 10.1080/09583157.2018.1531111

[B7] Castrillo L. Griggs M. Ranger C. Reding M. Vandenberg J. (2011). Virulence of commercial strains of *Beauveria bassiana* and *Metarhizium brunneum* (Ascomycota: Hypocreales) against adult *Xylosandrus germanus* (Coleoptera: Curculionidae) and impact on brood. *Biol. Control* 58 121–126. 10.1016/j.biocontrol.2011.04.010

[B8] Chen W. Han Y. Liang J. Liang Z. (2019). Morphological and phylogenetic characterization of novel *Metarhizium* species in Guizhou, China. *Phytotaxa* 419 189–196. 10.11646/phytotaxa.419.2.5

[B9] Chen W. Han Y. Liang J. Liang Z. Jin D. (2017). *Metarhizium dendrolimatilis*, a novel *Metarhizium* species parasitic on *Dendrolimus* sp. larvae. *Mycosphere* 8 31–37. 10.5943/mycosphere/8/1/4

[B10] Chen Z. Xu L. Yang X. Zhang Y. Yang Y. (2018). *Metarhizium baoshanense* sp. nov., a New entomopathogen fungus from Southwestern China. *Pak. J. Zool.* 50 1739–1746. 10.17582/journal.pjz/2018.50.5.1739.1746

[B11] Edler D. Klein J. Antonelli A. Silvestro D. (2019). raxmlGUI 2.0 beta: a graphical interface and toolkit for phylogenetic analyses using RAxML. *bioRxiv* [Preprint]. 10.1101/800912

[B12] Filizola P. R. B. Luna M. A. C. de Souza A. F. Coelho I. L. Laranjeira D. Campos-Takaki G. M. (2019). Biodiversity and phylogeny of novel *Trichoderma* isolates from mangrove sediments and potential of biocontrol against *Fusarium* strains. *Microb. Cell Fact.* 18:89. 10.1186/s12934-019-1108-y 31122261 PMC6532204

[B13] Frisvad J. Samson R. (2004). Polyphasic taxonomy of *Penicillium* subgenus *Penicillium*. A guide to identif cation of food and airborne terverticillate Penicillia and their mycotoxins. *Stud. Mycol.* 2004 1–173.

[B14] Gutierrez A. Leclerque A. Manfrino R. Luz C. Ferrari W. Barneche J. (2019). Natural occurrence in Argentina of a new fungal pathogen of cockroaches, *Metarhizium argentinense* sp. nov. *Fungal. Biol. U.K.* 123 364–372. 10.1016/j.funbio.2019.02.005 31053325

[B15] Hawksworth D. Lücking R. (2017). Fungal diversity revisited: 2.2 to 3.8 million species. *Microbiol. Spectr.* 5:FUNK-0052-2016. 10.1128/microbiolspec 28752818 PMC11687528

[B16] Hussain A. Rizwan-ul-Haq M. Al-Ayedh H. Ahmed S. Al-Jabr A. (2015). Effect of *Beauveria bassiana* infection on the feeding performance and antioxidant defence of red palm weevil, *Rhynchophorus ferrugineus*. *BioControl.* 60 849–859. 10.1007/s10526-015-9682-3

[B17] Hyde K. D. Xu J. Rapior S. Jeewon R. Lumyong S. Niego A. G. T. (2019). The amazing potential of fungi: 50 ways we can exploit fungi industrially. *Fungal Divers.* 97 1–136. 10.1007/s13225-019-00430-9

[B18] Hywel-Jones N. (1994). *Cordyceps khaoyaiensis* and *C. pseudomilitaris*, two new pathogens of lepidopteran larvae from Thailand. *Mycol. Res.* 98 939–942. 10.1016/S0953-7562(09)80267-0

[B19] Jeewon R. Hyde K. (2016). Establishing species boundaries and new taxa among fungi: recommendations to resolve taxonomic ambiguities. *Mycosp.* 7 1669–1677. 10.5943/mycosphere/7/11/4

[B20] Kepler R. Humber R. Bischoff J. Rehner S. (2014). Clarification of generic and species boundaries for *Metarhizium* and related fungi through multigene phylogenetics. *Mycologia* 106 811–829. 10.3852/13-319 24891418

[B21] Khan B. Ahmad K. Zafar J. Shoukat R. Farooq M. (2018). Efficacy of different entomopathogenic fungi on biological parameters of pulse beetle *Callosobruchus chinensis* L. (Coleoptera: Bruchidae). *J. Entomol. Zool. Stud.* 6 1972–1976.

[B22] Kumar S. Stecher G. Tamura K. (2016). MEGA7: molecular evolutionary genetics analysis version 7.0 for bigger datasets. *Mol Biol Evol.* 33 1870–1874. 10.1093/molbev/msw054 27004904 PMC8210823

[B23] Liang J. Liu B. Li Z. Meng W. Wang Q. Xu L. (2019). *Myxotrichum albicans*, a new slowly-growing species isolated from forest litters in China. *Mycoscience* 60 232–236. 10.1016/j.myc.2019.03.002

[B24] Luangsa-ard J. J. Mongkolsamrit S. Thanakitpipattana D. Khonsanit A. Tasanathai K. Noisripoom W. (2017). *Clavicipitaceous entomopathogens*: new species in *Metarhizium* and a new genus *Nigelia*. *Mycol. Prog.* 16 369–391. 10.1007/s11557-017-1277-1

[B25] Metchnikoff E. (1879). *Maladies des Hannetons Duble. Zapiski Imperatorskogo Obshchestua Sel’skago Khozyaistra Yuzhnoi Rossii.* Odessa: Elsevier, 17–50.

[B26] Meyling N. Eilenberg J. (2007). Ecology of the entomopathogenic fungi *Beauveria bassiana* and *Metarhizium anisopliae* in temperate agroecosystems: potential for conservation biological control. *Biol. Control.* 43 145–155. 10.1016/j.biocontrol.2007.07.007

[B27] Mongkolsamrit S. Khonsanit A. Thanakitpipattana D. Tasanathai K. Noisripoom W. Lamlertthon S. (2020). Revisiting *Metarhizium* and the description of new species from Thailand. *Stud. Mycol.* 95 171–251. 10.1016/j.simyco.2020.04.001 32855740 PMC7426330

[B28] Nishi O. Shimizu S. Sato H. (2017). *Metarhizium bibionidarum* and *M. purpureogenum*: new species from Japan. *Mycol. Prog.* 16 987–998. 10.1007/s11557-017-1333-x

[B29] Noble R. Dobrovin-Pennington A. Fitzgerald J. Dew K. Wilson C. Ross K. (2018). Improving biocontrol of black vine weevil (*Otiorhynchus sulcatus*) with entomopathogenic fungi in growing media by incorporating spent mushroom compost. *BioControl.* 63 697–706. 10.1007/s10526-018-9877-5

[B30] Rogerson C. T. (1970). The hypocrealean fungi (*Ascomycetes*, *Hypocreales*). *Mycologia* 62 865–910. 10.1080/00275514.1970.12019033 5486005

[B31] Ronquist F. Teslenko M. van der Mark P. Ayres D. L. Darling A. Höhna S. (2012). MrBayes 3.2: efficient Bayesian phylogenetic inference and model choice across a large model space. *Syst. Biol.* 61 539–542. 10.1093/sysbio/sys029 22357727 PMC3329765

[B32] Sajid M. Bashir N. Batool Q. Munir I. Ameen M. Munir S. (2017). In-vitro evaluation of biopesticides (*Beauveria bassiana*, *Metarhizium anisopliae*, *Bacillus thuringiensis*) against mustard aphid *Lipaphis erysimi* kalt. (Hemiptera: Aphididae). *J. Entomol. Zool. Stud.* 5 331–335.

[B33] Schardl C. L. Young C. A. Hesse U. Amyotte S. G. Andreeva K. Calie P. J. (2013). Plant-symbiotic fungi as chemical engineers: multi-genome analysis of the clavicipitaceae reveals dynamics of alkaloid loci. *PLoS Genet.* 9:e1003323. 10.1371/journal.pgen.1003323 23468653 PMC3585121

[B34] Sung G. H. Hywel-Jones N. L. Sung J. M. Luangsa-ard J. J. Shrestha B. Spatafora J. W. (2007a). Phylogenetic classification of *Cordyceps* and the clavicipitaceous fungi. *Stud. Mycol.* 57 5–59. 10.3114/sim.2007.57.01 18490993 PMC2104736

[B35] Sung G. H. Sung J. M. Hywel-Jones N. L. Spatafora J. W. (2007b). A multi-gene phylogeny of *Clavicipitaceae* (Ascomycota, Fungi): identification of localized incongruence using a combinational bootstrap approach. *Mol. Phylogenet. Evol.* 44 1204–1223. 10.1016/j.ympev.2007.03.011 17555990

[B36] Tulloch M. (1976). The genus *Metarhizium*. *Trans. Br. Mycol. Soc.* 66 407–411. 10.1016/S0007-1536(76)80209-4

[B37] Tzean S. Hsieh L. Chen J. Wu W. (1993). *Nomuraea cylindrospora* comb. Nov. *Mycologia* 85 514–519. 10.1080/00275514.1993.12026302

[B38] Wijayawardene N. N. Hyde K. D. Dai D. Q. Tang L. Z. Aptroot A. Castañeda-Ruiz R. F. (2020). A dynamic portal for a community-driven, continuously updated classification of Fungi and fungus-like organisms: outlineoffungi.org. *Mycosphere* 11 1514–1526. 10.5943/mycosphere/11/1/11

[B39] Willis K. J. (ed.) (2018). *State of the World’s Fungi 2018. Report.* London: Royal Botanic Gardens.

[B40] Wu S. Sarkar S. Lv J. Xu X. Lei Z. (2020). Poor infectivity of *Beauveria bassiana* to eggs and immatures causes the failure of suppression on *Tetranychus urticae* population. *BioControl* 65 81–90. 10.1007/s10526-019-09970-0

[B41] Zimmermann G. (1993). The entornopathogenic fungus *Metarhizium anisopliae* and its potential as a biocontrol agent. *Pestic. Sci.* 37 375–379. 10.1002/ps.2780370410

[B42] Zimmermann G. (2007). Review on safety of the entomopathogenic fungus *Metarhizium anisopliae*. *Biocontrol Sci. Techn.* 17 879–920. 10.1080/09583150701593963

