# Supplementary material for: Parametarhizium (Clavicipitaceae) gen. nov. With Two New Species as a Potential Biocontrol Agent Isolated From Forest Litters in Northeast China
Source: Front Microbiol. 2021 Feb 19;12:627744. doi: 10.3389/fmicb.2021.627744 (PMC7933043; doi:10.3389/fmicb.2021.627744)
Supplement: Supplementary file 1 [file Data_Sheet_1.docx]

**Supplementary file**

**Supplementary Figure Legends**

**Figure S1.** Phylogenetic analysis of *Parametarhizium* and 34 genera in *Clavicipitaceae.*

**Figure S2.** Colonies of *Parametarhizium changbaiense* and *Parametarhizium hingganense* on different media. **(A-C)** *P. changbaiense* colonies (front left side, reverse right side) on Sabouraud dextrose agar with yeast extract (SDAY), Malt extract powder (MEA), and Oatmeal agar (OA). **(D-F)** *P. hingganense* colonies (front left side, reverse right side) on SDAY, MEA, and OA.

**Figure S3.** Cadavers caused by *Parametarhizium changbaiense* and *Parametarhizium hingganense*. **(A,B)** *Monolepta hieroglyphica* and *Callosobruchus chinensis* infected by *P. changbaiense*. **(C,D)** *Mo. hieroglyphica* and *C. chinensis* infected by *P. hingganense*.

**Supplementary Table list**

**Table S1.** Primers information for 7 DNA sequences

**Table S2.** List of specimens and GenBank accession numbers of sequences used in this study

**Table S3.** Sequence similarity analyses of between *Parametarhizium changbaiense* and its closest fungal species in GenBank

**Table S4.** Sequence similarity analyses of between *Parametarhizium hingganense* and its closest fungal species in GenBank

**Table S5.** Average conidial sizes of *Parametarhizium changbaiense* and *Parametarhizium hingganense* on different media

**
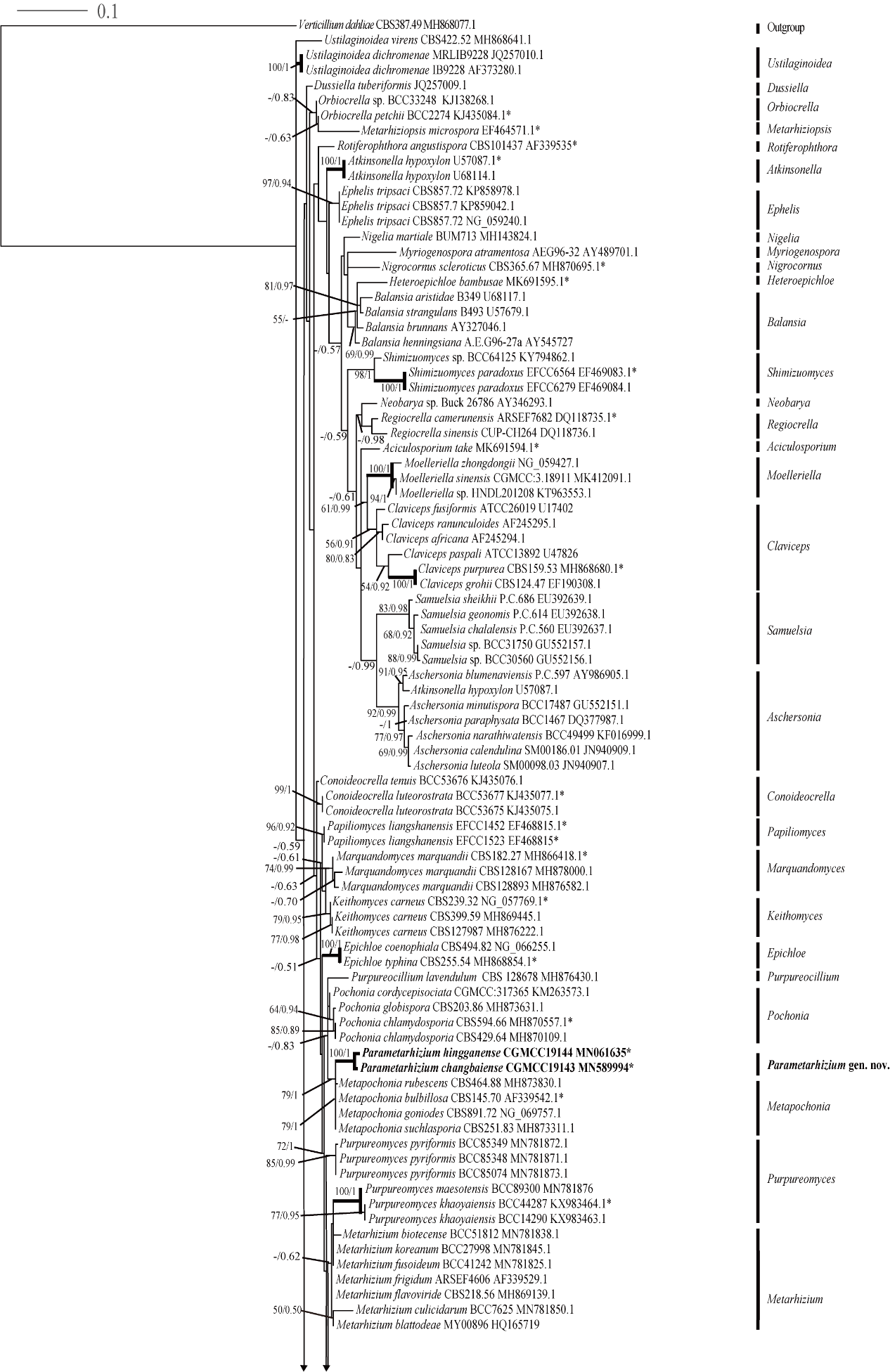
**

**
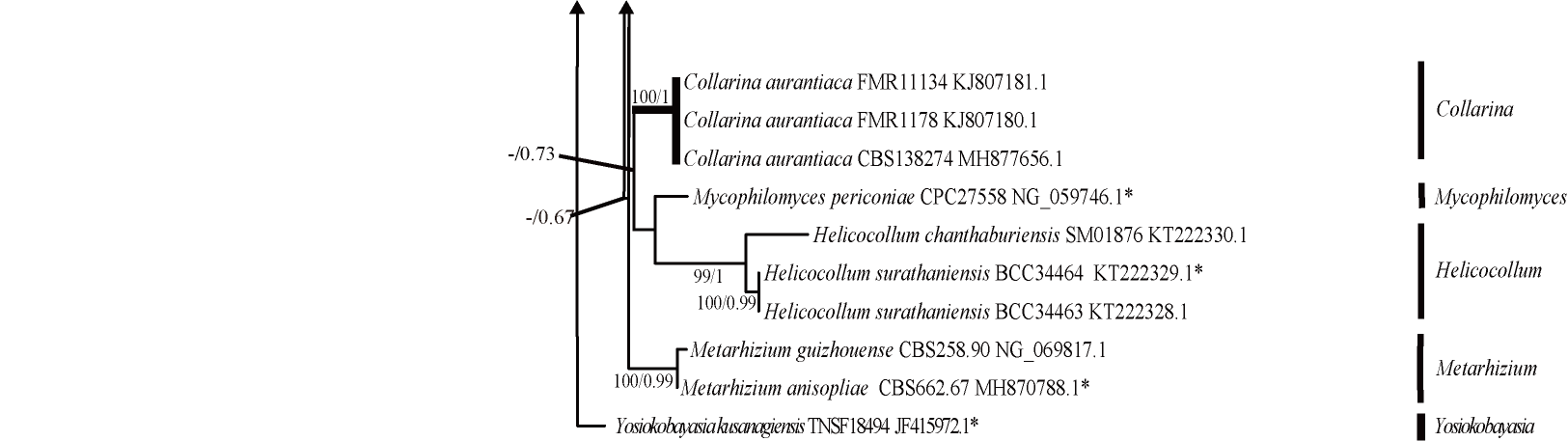
**

**Figure S1.** Phylogenetic analysis of *Parametarhizium* and 34 genera in *Clavicipitaceae.* Phylogenetic based on nuclear large subunit rDNA (LSU) using maximum likelihood as optimality criterion. ML bootstrap values / Bayesian posterior probability above 50% (MLBS)/0.5 (BPP) are shown on the nodes. *ex-type material.

**Description**

The family *Clavicipitaceae* (*Ascomycota*, *Hypocreales*) is a large group of fungi with 48 genera (including 6 new genera erected in 2020). To include as many genera in *Clavicipitaceae* as possible, a phylogenetic analysis (Supplementary Figure S1) only based on the LSU sequences from 34 genera in *Clavicipitaceae* was carried out. The LSU sequences of the other 14 genera are not available. The phylogenetic analysis of aligned LSU sequences used maximum-likelihood algorithm by RAxML including 1000 bootstrap replicates. A general time-reversible model (GTR) with a gamma-distributed rate variation was used in the maximum-likelihood analyses. Markov chain Monte Carlo (MCMC) was used to estimate posterior probability by MrBayes v. 3.2.4. Four simultaneous Markov chains were run for 1,000,000 generations (standard deviation of split frequencies less than 0.01) and trees were sampled every 1000 generations. The first 2000 trees representing the burn-in phase of the analysis were discarded and the remaining trees were used to determine posterior probabilities in the majority rule consensus tree.

This phylogenetic analysis based on the LSU sequences also showed the *Parametarhizium* species (SGSF125 and SGSF355) formed a monophyletic clade whose closest clade is genus *Metapochonia* (belonging to formerly *Metarhizium sensu lato*). To infer their phylogenetic relationships between *Parametarhizium* and *Metarhizium sensu lato*, a six-gene dataset consisting of DNA fragments of [Nuclear small subunit rDNA (SSU) + LSU + TUB + TEF + RPB1 + RPB2] were used for phylogenetic analysis as shown in Figure 3.


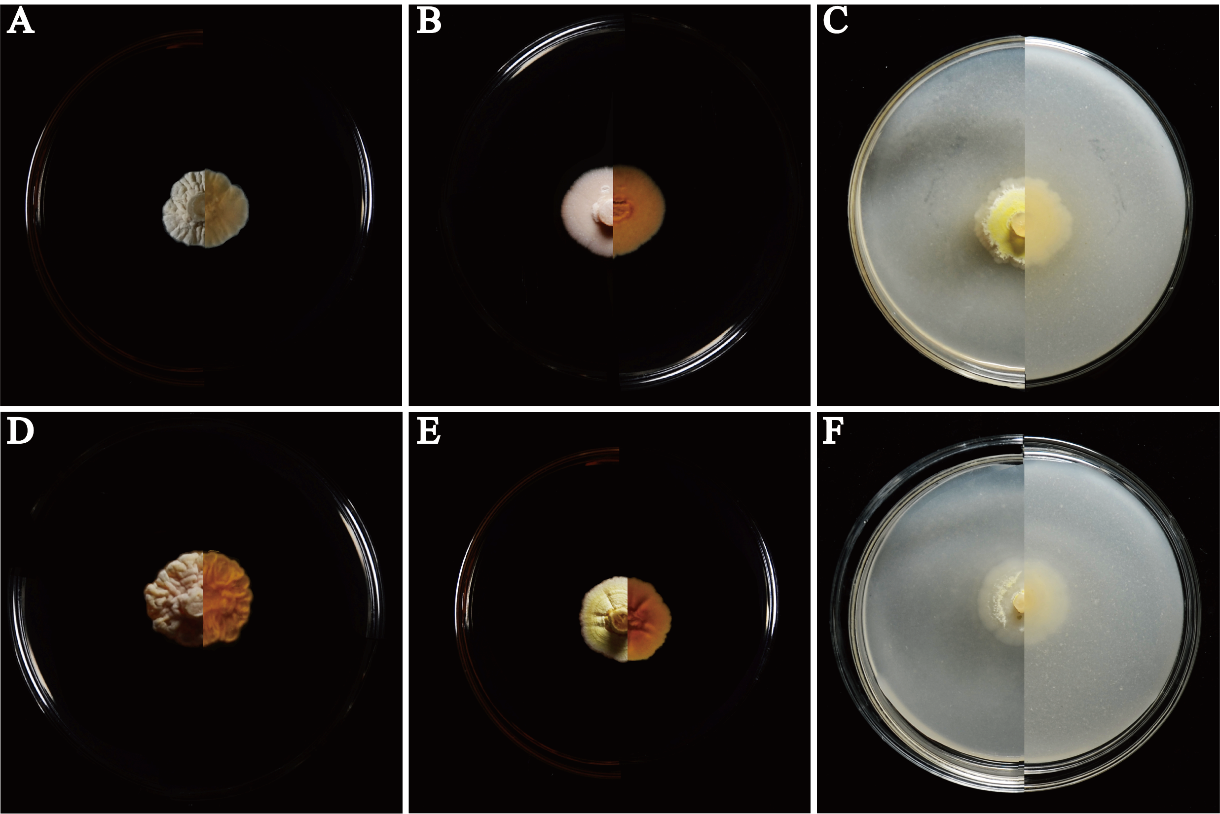


**Figure S2.** Colonies of *Parametarhizium changbaiense* and *Parametarhizium hingganense* on different media. **(A-C)** *P. changbaiense* colonies (front left side, reverse right side) on Sabouraud dextrose agar with yeast extract (SDAY), Malt extract powder (MEA), and Oatmeal agar (OA). **(D-F)** *P. hingganense* colonies (front left side, reverse right side) on SDAY, MEA, and OA.

**Description**

The colonies of *P. changbaiense* on different media are shown in Supplementary Figure S2 a-c. Colonies on SDAY (Supplementary Figure S2 a) reaching 15–17 mm, pale yellow, wrinkled, velvety, with undulate margin; reverse yellow. Colonies on MEA (Supplementary Figure S2 b) reaching 15–18 mm, white, flat; reverse brown. Colonies on OA (Supplementary Figure S2 c) reaching 18–19 mm, flat, initially white, turning lemon–yellow due to the production of conidial masses, exudate lemon yellow; reverse pale yellow.

The colonies of *P. changbaiense* on different media are shown in Supplementary Figure S2 d-f. Colonies on SDAY (Supplementary Figure S2 d) reaching 17–20 mm, pale yellow, wrinkled, velvety, with undulate margin; reverse brown, wrinkled. Colonies on MEA (Supplementary Figure S2 e) reaching 15–18 mm, lemon yellow, radially sulcate, velvety; reverse brown, radially sulcate. Colonies on OA (Supplementary Figure S2 f) reaching 18–19 mm, flat, initially cream, turning lemon–yellow due to the production conidial masses; reverse pale yellow.


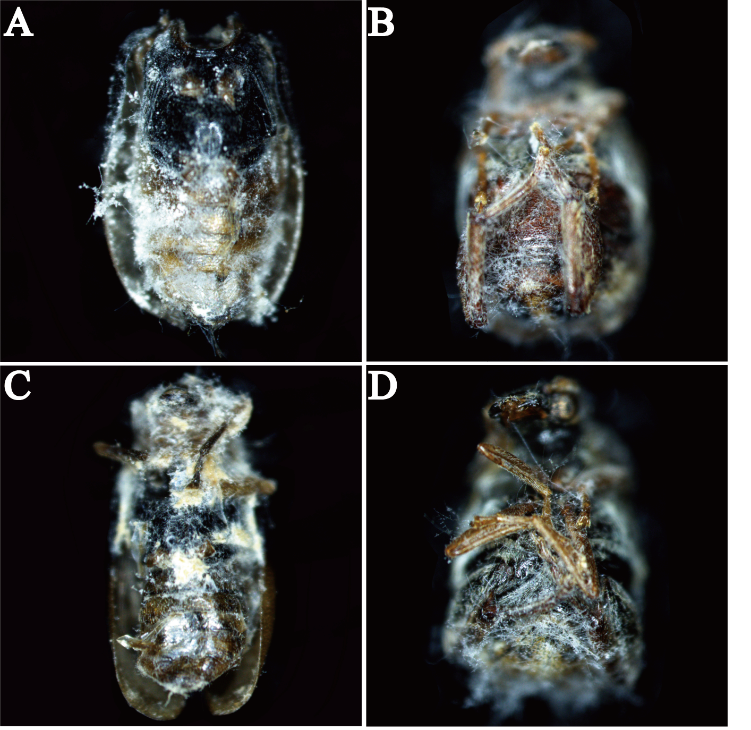


**Figure S3.** Cadavers caused by *Parametarhizium changbaiense* and *Parametarhizium hingganense*. **(A,B)** *Monolepta hieroglyphica* and *Callosobruchus chinensis* infected by *P. changbaiense*. **(C,D)** *Mo. hieroglyphica* and *C. chinensis* infected by *P. hingganense*.

**Description**

The external white mycelia of *P. changbaiense* and *P. hingganense* on the cadavers of *Mo. hieroglyphica* and *C. chinensis* firstly emerged from the abdomen, and then gradually surrounded the insects. In contrast to green cadavers caused by most *Metarhizium* spp. such as *M. anisopliae* and *M. robertsii*, the colors of cadavers caused by *P. changbaiense* and *P. hingganense* were white, respectively (Supplementary Figure S3).

**Table S1.** Primers information for 7 DNA sequences

| Name | Length | Direction | Sequence 5ˊ-3ˊ |
| --- | --- | --- | --- |
| ITS1 | 19 | forward | TCCGTAGGTGAACCTGCGG |
| ITS4 | 20 | reverse | TCCTCCGCTTATTGATATGC |
| LR0R | 17 | forward | ACCCGCTGAACTTAAGC |
| LR5 | 17 | reverse | TCCTGAGGGAAACTTCG |
| NS1 | 19 | forward | GTAGTCATATGCTTGTCTC |
| NS4 | 20 | reverse | CTTCCGTCAATTCCTTTAAG |
| EF1-983F | 23 | forward | GCYCCYGGHCAYCGTGAYTTYAT |
| EF1-2218R | 23 | reverse | ATGACACCRACRGCRACRGTYTG |
| Bt2a | 24 | forward | GGTAACCAAATCGGTGCTGCTTTC |
| Bt2b | 24 | reverse | ACCCTCAGTGTAGTGACCCTTGGC |
| RPB1-Af | 20 | forward | GARTGYCCDGGDCAYTTYGG |
| RPB1-VH6R | 24 | reverse | ATGACCCATCATRGAYTCCTTRTG |
| fRPB2-5f | 20 | forward | GAYGAYMGWGATCAYTTYGG |
| fRPB2-7cR | 20 | reverse | CCCATRGCTTGYTTRCCCAT |

**Description**

The following 7 DNA sequences containing ITS, SSU, LSU, TUB, TEF, RPB1, and RPB2 were amplified by PCR. The primers for the above sequences are shown in Supplementary Table S1, respectively.

**Table S2.** List of specimens and GenBank accession numbers of sequences used in this study

| **Species** | **Collection** | **SSU** | **LSU** | **TUB** | **TEF** | **RPB1** | **RPB2** |
| --- | --- | --- | --- | --- | --- | --- | --- |
| *Metarhizium album* | ARSEF 1941 | - | - | EU248840 | EU248868 | EU248920 | EU248948 |
| *Metarhizium album* | ARSEF 1942 | - | - | KJ398572 | KJ398802 | KJ398611 | KJ398709 |
| *Metarhizium album* | ARSEF 2082 | DQ522560 | - | KJ398579 | DQ522352 | KJ398617 | KJ398715 |
| *Metarhizium album* | ARSEF 2179 | - | - | KJ398580 | KJ398807 | KJ398618 | KJ398716 |
| *Metarhizium acridum* | ARSEF 324 | - | - | EU248812 | EU248844 | EU248896 | EU248924 |
| *Metarhizium acridum* | ARSEF 7486 | - | - | EU248813 | EU248845 | EU248897 | EU248925 |
| [*Metarhizium alvesii*](http://www.speciesfungorum.org/Names/GSDSpecies.asp?RecordID=819472) | ARSEF 13308 | - | - | KY007611 | KY007614 | KY007612 | KY007613 |
| [*Metarhizium anisopliae*](http://www.speciesfungorum.org/Names/GSDSpecies.asp?RecordID=101834) | ARSEF 7450 | - | - | EU248823 | EU248852 | EU248904 | EU248932 |
| [*Metarhizium anisopliae*](http://www.speciesfungorum.org/Names/GSDSpecies.asp?RecordID=101834) | ARSEF 7487 | - | - | EU248822 | DQ463996 | DQ468355 | DQ468370 |
| [*Metarhizium argentinense*](http://www.speciesfungorum.org/Names/GSDSpecies.asp?RecordID=822918) | ARSEF 13510 | - | - | MF966623 | MF966624 | MF966625 | MF966622 |
| *Metarhizium biotecense* | BCC 51812 | MN781937 | MN781838 | - | MN781693 | MN781745 | MN781792 |
| [*Metarhizium blattodeae*](http://www.speciesfungorum.org/Names/GSDSpecies.asp?RecordID=815131) | MY00896 | HQ165657 | HQ165719 | KU182914 | HQ165678 | HQ165739 | HQ165638 |
| [*Metarhizium brasiliense*](http://www.speciesfungorum.org/Names/GSDSpecies.asp?RecordID=808035) | ARSEF 2948b | - | - | KJ398582 | KJ398809 | KJ398620 | KJ398718 |
| [*Metarhizium brunneum*](http://www.speciesfungorum.org/Names/GSDSpecies.asp?RecordID=259046) | ARSEF 2107 | - | - | EU248826 | EU248855 | EU248907 | EU248935 |
| *Metarhizium candelabrum* | BCC 29224 | MN781952 | MN781853 | - | MN781708 | MN781755 | MN781804 |
| *Metarhizium cercopidarum* | BCC 31660 | MN781953 | BCC31660 | - | MN781709 | MN781756 | MN781805 |
| *Metarhizium cicadae* | BCC 48696 | MN781948 | MN781848 | - | MN781703 | - | MN781800 |
| *Metarhizium clavatum* | BCC 84543 | - | MN781834 | - | MN781689 | MN781741 | MN781789 |
| *Metarhizium culicidarum* | BCC 7600 | MN781951 | MN781852 | - | MN781707 | MN781754 | MN781803 |
| *Metarhizium cylindrosporum* | ARSEF 6926 | - | - | KJ398587 | KJ398783 | KJ398543 | KJ398723 |
| *Metarhizium cylindrosporum* | CBS 256.90b | - | - | KJ398543 | KJ398814 | KJ398594 | KJ398691 |
| *Metarhizium ebumeum* | BCC 79252 | - | MN781829 | - | MN781682 | MN781736 | - |
| *Metarhizium ellipsoideum* | BCC 49285 | MN781957 | MN781858 | - | MN781713 | MN781759 | MN781808 |
| [*Metarhizium flavoviride*](http://www.speciesfungorum.org/Names/GSDSpecies.asp?RecordID=317596) | CBS 218.56b | - | - | KJ398555 | KJ398787 | KJ398598 | KJ398694 |
| [*Metarhizium flavoviride*](http://www.speciesfungorum.org/Names/GSDSpecies.asp?RecordID=317596) | ARSEF 2025 | - | - | KJ398575 | KJ398804 | KJ398614 | KJ398712 |
| *Metarhizium flavum* | BCC 90870 | MN781965 | MN781874 | - | MN781731 | MN781776 | MN781822 |
| [*Metarhizium frigidum*](http://www.speciesfungorum.org/Names/GSDSpecies.asp?RecordID=501374) | ARSEF 4124 | - | - | EU248828 | DQ464002 | DQ468361 | DQ468376 |
| [*Metarhizium frigidum*](http://www.speciesfungorum.org/Names/GSDSpecies.asp?RecordID=501374) | ARSEF 7445 | - | - | KJ398590 | KJ398818 | KJ398628 | KJ398727 |
| *Metarhizium fusoideum* | BCC 28246 | MN781944 | MN781844 | - | MN781699 | MN781749 | MN781796 |
| [*Metarhizium globosum*](http://www.speciesfungorum.org/Names/GSDSpecies.asp?RecordID=512408) | ARSEF 2596 | - | - | EU248814 | EU248846 | EU248898 | EU248926 |
| [*Metarhizium granulomatis*](http://www.speciesfungorum.org/Names/GSDSpecies.asp?RecordID=806081) | UAMH 11028 | HM635076 | HM195304 | - | KJ398781 | - | - |
| *Metarhizium gryllidicola* | BCC 37918 | - | MN781836 | - | MN781691 | MN781743 | MN781790 |
| *Metarhizium huainamdangense* | BCC 32190 | MN781954 | MN781855 | - | MN781710 | MN781757 | - |
| [*Metarhizium indigoticum*](http://www.speciesfungorum.org/Names/GSDSpecies.asp?RecordID=806083) | TNS-F 18553 | JF415953 | JF415968 | KJ398569 | JF416010 | JN049886 | JF415992 |
| [*Metarhizium indigoticum*](http://www.speciesfungorum.org/Names/GSDSpecies.asp?RecordID=806083) | NBRC 100684 | JF415952 | JF415969 | KJ398544 | KJ398784 | KJ398595 | KJ398692 |
| *Metarhizium* [*kalasinense*](http://www.speciesfungorum.org/Names/GSDSpecies.asp?RecordID=808402) | BCC 12687 | - | - | KJ398564 | KJ398796 | JN049888 | KJ398703 |
| [*Metarhizium koreanum*](http://www.speciesfungorum.org/Names/GSDSpecies.asp?RecordID=808036) | ARSEF 2038 | - | - | KJ398577 | KJ398805 | KJ398615 | KJ398713 |
| [*Metarhizium koreanum*](http://www.speciesfungorum.org/Names/GSDSpecies.asp?RecordID=808036) | ARSEF 2039 | - | - | KJ398578 | KJ398806 | KJ398616 | KJ398714 |
| [*Yosiokobayasia kusanagiense*](http://www.speciesfungorum.org/Names/GSDSpecies.asp?RecordID=806085) | TNS-F 18494 | JF415954 | JF415972 | KJ398568 | JF416014 | JN049890 | KJ398755 |
| [*Metarhizium lepidiotae*](http://www.speciesfungorum.org/Names/GSDSpecies.asp?RecordID=512409) | ARSEF 7412 | - | - | EU248836 | EU248864 | EU248916 | EU248944 |
| [*Metarhizium majus*](http://www.speciesfungorum.org/Names/GSDSpecies.asp?RecordID=512410) | ARSEF 1914b | - | - | KJ398571 | KJ398801 | KJ398610 | KJ398708 |
| [*Metarhizium majus*](http://www.speciesfungorum.org/Names/GSDSpecies.asp?RecordID=512410) | ARSEF 1946 | - | - | EU248839 | EU248867 | EU248919 | EU248947 |
| *Metarhizium megapomponiae* | BCC 25100 | MN781947 | MN781847 | - | MN781702 | MN781751 | MN781799 |
| [*Metarhizium minus*](http://www.speciesfungorum.org/Names/GSDSpecies.asp?RecordID=806092) | ARSEF 1764 | - | - | KJ398570 | KJ398800 | KJ398609 | KJ398707 |
| [*Metarhizium minus*](http://www.speciesfungorum.org/Names/GSDSpecies.asp?RecordID=806092) | ARSEF 2037b | AF339580 | AF339531 | KJ398576 | DQ522353 | DQ522400 | DQ522454 |
| *Metarhizium nomnoi* | BCC 19364 | MN781940 | MN781841 | - | MN781696 | MN781747 | - |
| [*Metarhizium novozealandicum*](http://www.speciesfungorum.org/Names/GSDSpecies.asp?RecordID=806093) | ARSEF 4661 | - | - | KJ398584 | KJ398810 | KJ398622 | KJ398720 |
| [*Metarhizium novozealandicum*](http://www.speciesfungorum.org/Names/GSDSpecies.asp?RecordID=806093) | ARSEF 4674 | - | - | KJ398585 | KJ398811 | KJ398623 | KJ398721 |
| *Metarhizium ovoidosporum* | BCC 32600 | MN781961 | MN781862 | - | MN781717 | MN781763 | - |
| *[Metarhizium owariense](http://www.speciesfungorum.org/Names/GSDSpecies.asp?RecordID=806094)* | NBRC 33258 | - | HQ165730 | KJ398545 | JF416017 | KJ398596 | JF415996 |
| [*Metarhizium pemphigi*](http://www.speciesfungorum.org/Names/GSDSpecies.asp?RecordID=807860) | ARSEF 6569 | - | - | KJ398586 | KJ398813 | KJ398624 | KJ398722 |
| [*Metarhizium pemphigi*](http://www.speciesfungorum.org/Names/GSDSpecies.asp?RecordID=807860) | ARSEF 7491 | - | - | KJ398591 | KJ398819 | KJ398629 | KJ398728 |
| *Metarhizium phasmatodeae* | BCC 2841 | - | MN781828 | - | MN781681 | MN781738 | MN781782 |
| *Metarhizium phuwaiangense* | BCC 78206 | - | - | - | MN781719 | MN781765 | MN781812 |
| *Metarhizium phuwaiangense* | BCC 85069 | - | MN781865 | - | MN781721 | MN781767 | MN781814 |
| *Metarhizium purpureonigrum* | BCC 89249 | MN781963 | MN781869 | - | MN781726 | MN781772 | MN781817 |
| *Metarhizium purpureum* | BCC 82642 | - | MN781867 | - | MN781723 | MN781769 | MN781816 |
| [*Metarhizium pseudoatrovirens*](http://www.speciesfungorum.org/Names/GSDSpecies.asp?RecordID=806096) | NBRC 103797 | - | JF415977 | KJ398546 | KJ398785 | JN049893 | JF415997 |
| [*Metarhizium rileyi*](http://www.speciesfungorum.org/Names/GSDSpecies.asp?RecordID=807862) | ARSEF 936 | - | - | KJ398566 | KJ398798 | KJ398607 | KJ398705 |
| [*Metarhizium robertsii*](http://www.speciesfungorum.org/Names/GSDSpecies.asp?RecordID=512411) | ARSEF 727 | - | - | EU248816 | DQ463994 | DQ468353 | DQ468368 |
| [*Metarhizium robertsii*](http://www.speciesfungorum.org/Names/GSDSpecies.asp?RecordID=512411) | ARSEF 7501 | - | - | EU248818 | EU248849 | EU248901 | EU248929 |
| *Metarhizium samlanense* | BCC 17091 | HQ165665 | HQ165727 | - | HQ165686 | - | HQ165646 |
| *Metarhizium sulphureum* | BCC 39045 | MK632120 | - | - | MN781686 | - | - |
| [*Metarhizium viride*](http://www.speciesfungorum.org/Names/GSDSpecies.asp?RecordID=806098) | ARSEF 2456 | - | - | KJ398581 | KJ398808 | KJ398619 | KJ398717 |
| [*Metarhizium viridulum*](http://www.speciesfungorum.org/Names/GSDSpecies.asp?RecordID=487851) | BCC 36261 | MN781930 | MN781827 | - | MN781680 | MN781737 | MN781781 |
| *Metarhizium viridulum* | ARSEF 6927 | - | - | - | KJ398815 | KJ398681 | - |
| *Metapochonia bulbillosa* | CBS 145.70 | AF339591 | - | KJ398549 | EF468796 | EF468902 | EF468943 |
| *Metapochonia bulbillosa* | CBS 247.68 | - | - | KJ398556 | KJ398788 | AB758663 | KJ398695 |
| *Metapochonia goniodes* | CBS 891.72 | AF339599 | - | KJ398551 | DQ522354 | DQ522401 | DQ522458 |
| *Metapochonia microbactrospora* | CBS 101433 | - | - | KJ398562 | KJ398794 | KJ398605 | KJ398701 |
| *Metapochonia rubescens* | CBS 464.88 | - | - | KJ398552 | EF468797 | EF468903 | EF468944 |
| *Metapochonia rubescens* | CBS 110436 | - | - | KJ398563 | KJ398795 | KJ398606 | KJ398702 |
| *Metapochonia suchlasporia* | CBS 248.83 | - | - | KJ398557 | KJ398789 | KJ398600 | KJ398696 |
| *Metapochonia suchlasporia* | CBS 814.83 | - | - | KJ398561 | KJ398793 | KJ398604 | KJ398700 |
| *Metapochonia suchlasporia* | CBS 251.83 | - | - | KJ398558 | KJ398790 | KJ398601 | KJ398697 |
| *Pochonia chlamydosporia* | CBS 504.66 | - | - | KJ398550 | EF469069 | EF469098 | EF469120 |
| *Pochonia chlamydosporia* | CBS 103.65 | - | - | KJ398554 | KJ398786 | KJ398597 | KJ398693 |
| *Pochonia chlamydosporia* | CBS 429.64 | - | - | KJ398559 | KJ398791 | KJ398602 | KJ398698 |
| *Pochonia chlamydosporia* | CBS 594.66 | - | - | KJ398560 | KJ398792 | KJ398603 | KJ398699 |
| *Keithomyces sp.* | CBS 126563 | MT078871 | MT078856 | - | - | MT078864 | MT078921 |
| *Keithomyces carneus* | CBS 239.32 | - | NG_057769 | KJ398547 | EF468789 | EF468894 | EF468938 |
| [*Purpureomyces khaoyaiense*](http://www.speciesfungorum.org/Names/GSDSpecies.asp?RecordID=806084) | BCC 14290 | JF415970 | - | KJ398565 | KJ398797 | JN049889 | KJ398704 |
| *Purpureomyces maesotensis* | BCC 89300 | - | MN781876 | - | MN781733 | MN781778 | - |
| *Purpureomyces pyriformis* | BCC 85074 | - | MN781873 | - | MN781730 | MN781775 | MN781821 |
| *Marquandomyces sp.* | CBS 127132 | MT078872 | MT078857 | - | - | MT078865 | MT078922 |
| [*Marquandomyces marquandii*](http://www.speciesfungorum.org/Names/GSDSpecies.asp?RecordID=806091) | CBS 182.27 | EF468990 | EF468845 | KJ398548 | EF468793 | EF468899 | EF468942 |
| [*Sungia yongmunense*](http://www.speciesfungorum.org/Names/GSDSpecies.asp?RecordID=806097) | EFCC 2131 | - | - | KJ398542 | EF468770 | EF468876 | KJ398690 |
| *Rotiferophthora angustispora* | CBS 101437 | AF339584 | AF339535 | - | AF543776 | DQ522402 | DQ522460 |
| ***Parametarhizium changbaiense*** | **CGMCC 19143** | [**MN590231**](https://www.ncbi.nlm.nih.gov/nuccore/MN590231) | **MN589994** | **MT921830** | **MN908589** | **MN917168** | **MT921829** |
| ***Parametarhizium hingganense*** | **CGMCC 19144** | **MN055706** | **MN061635** | **MN061672** | **MN065770** | **MN917170** | **MT939494** |
| *Claviceps fusiformis* | ATCC 26019 | DQ522539 | U17402 | - | DQ522320 | DQ522366 | - |
| *Claviceps paspali* | ATCC 13892 | U32401 | U47826 | - | DQ522321 | DQ522367 | DQ522416 |
| *Cordyceps chlamydosporia* | CBS 101244 | - | DQ518758 | - | - | DQ522372 | - |
| *Hypocrella schizostachyi* | BCC14123 | DQ522557 | DQ518771 | - | DQ522346 | DQ522392 | DQ522447 |
| *Shimizuomyces paradoxus* | EFCC 6279 | EF469131 | EF469083 | - | - | EF469100 | - |
| *Torrubiella luteorostrata* | NHJ 12516 | EF468995 | EF468850 | - | EF468800 | EF468905 | - |
| *Torrubiella tenuis* | NHJ 6293 | EU369112 | EU369044 | - | EU369029 | EU369068 | EU369087 |
| *Chamaeleomyces viridis* | CBS 659.71 | HQ165673 | HQ165735 | - | HQ165692 | - | HQ165652 |
| *Papiliomyces liangshanensis* | EFCC 1523 | EF468961 | EF468815 | - | EF468755 | - | - |
| *Papiliomyces shibinensis* | GZUHSB13050311 | KR153588 | - | - | KR153589 | KR153590 | - |
| *Metacordyceps neogunnii* | BUM 415 | MH143845 | - | - | MH143861 | MH143876 | - |
| *Balansia pilulaeformis* | A.E.G 94-2 | AF543764 | AF543788 | - | DQ522319 | DQ522365 | DQ522414 |
| *Balansia henningsiana* | A.E.G 96-27a | AY545723 | AY545727 | - | AY489610 | AY489643 | DQ522413 |
| *Regiocrella carmerunensis* | ARSEF 7682 | - | DQ118735 | - | DQ118743 | DQ127234 | - |
| *Petchia siamensis* | BCC 68420 | MK632113 | MK632087 | - | - | MK632163 | MK632140 |

**Description**

All available sequences of the molecular taxonomic markers from the two new and closely related species (*Metarhizium* and the genera related to *Metarhizium*) were combined for a multigene phylogenetic analysis (Supplementary Table S2). The combined dataset with concatenated a six-gene dataset consisting of DNA fragments of the SSU 735 bp, LSU 627 bp, TUB 253 bp, TEF 766 bp, RPB1 492 bp, and RPB2 778 bp. Sequences of the genus *Hypocrella* was used as outgroup.

**Table S3.** Sequence similarity analyses of between *Parametarhizium changbaiense* and its closest fungal species in GenBank

| Sequence | Closest strain | Similarity (%) | Cover (%) |
| --- | --- | --- | --- |
| ITS | *Keithomyces aciculare* | 91.67 | 100 |
| SSU | *Marquandomyces marquandii* | 99.34 | 100 |
| LSU | *Metarhizium flavoviride* | 97.44 | 100 |
| TUB | *Metapochonia* *suchlasporia* | 80.92 | 99 |
| TEF | *Metarhizium flavoviride* | 95.50 | 100 |
| RPB1 | *Metarhizium frigidum* | 84.95 | 97 |
| RPB2 | *Metarhizium flavoviride* | 82.47 | 98 |

**Description**

The sequence similarity (Supplementary Table S3) of between *Parametarhizium changbaiense* and the closest fungal species in GenBank were from 80.92% (TUB) to 99.34% (SSU) by BLAST program (http://blast.ncbi.nlm.nih.gov/). According to the similarity, the closest species were *Metapochonia suchlasporia*, *K. aciculare*, *Ma. marquandii,* and two *Metarhizium* spp. including, *M. flavoviride*, and *M. frigidum.* The morphological differences between *P. changbaiense* and other species were compared in the main text.

**Table S4.** Sequence similarity analyses of between *Parametarhizium hingganense* and its closest fungal species in GenBank

| Sequence | Closest strain | Similarity (%) | Cover (%) |
| --- | --- | --- | --- |
| ITS | *Keithomyces carneum* | 92.66 | 100 |
| SSU | *Marquandomyces marquandii* | 99.34 | 100 |
| LSU | *Papiliomyces liangshanensis* | 97.56 | 100 |
| TUB | *Keithomyces carneum* | 80.64 | 100 |
| TEF | *Metarhizium globosum* | 90.01 | 100 |
| RPB1 | *Metarhizium flavoviride* | 83.63 | 95 |
| RPB2 | *Metarhizium blattodeae* | 83.32 | 99 |

**Description**

The sequence similarity (Supplementary Table S4) of between *Parametarhizium hingganense* and the closest fungal species in GenBank were from 80.64% (TUB) to 99.34% (SSU) by BLAST program (http://blast.ncbi.nlm.nih.gov/). According to the similarity, the closest species were *Papiliomyces liangshanensis*, *K. carneum*, *Ma. marquandii*, and two *Metarhizium* spp. including *M. flavoviride*, and *M. blattodeae.* The morphological differences between *P. hingganense* and other species were compared in the main text.

**Table S5.** Average conidial sizes of *Parametarhizium changbaiense* and *Parametarhizium hingganense* on different media

| Strains No. | SDAY | MEA | OA |
| --- | --- | --- | --- |
| *Parametarhizium changbaiense* | 1.9 × 1.4 μm | 1.8 × 1.5 μm | 1.9 × 1.5 μm |
| *Parametarhizium hingganense* | 1.7 × 1.4 μm | 1.8 × 1.5 μm | 1.7 × 1.5 μm |

**Description**

Conidial sizes of *P. changbaiense* and *P. hingganense* on SDAY, MEA, and OA were measured (Supplementary Table S5). Their conidial sizes are close and the conidial sizes of *P. hingganense* are slightly smaller than *P. changbaiense*. Both of them are smaller < 3.3 μm than that most of *Metarhizium* spp. and other related species are bigger than 3.3 μm.

**Sequences of *Parametarhizium changbaiense* and *P. hingganense* in FASTA format**

*>**Parametarhizium changbaiense* SGSF125 SSU

TAGTATATTACTACTTGGATAACCGTGGTAATTCTAGAGCTAATACATGCTACAAATCCCGACTTCGGAAGGGATGTATTTATTAGATTAAAAACCAATGCCCTCTGGGCTCCTTGGTGACTCATGATAACTTCTCGAATCGCATGGCCTTGCGCCGGCGATGGTTCATTCAAATTTCTTCCCTATCAACTTTCGATGTTTGGGTAGTGGCCAAACATGGTTACAACGGGTAACGGAGGGTTAGGGCTCGACCCCGGAGAAGGAGCCTGAGAAACGGCTACTACATCCAAGGAAGGCAGCAGGCGCGCAAATTACCCAATCCCGATTCGGGGAGGTAGTGACAATAAATACTGATACAGGGCTCTTTTGGGTCTTGTAATTGGAATGAGTACAATTTAAATCCCTTAACGAGGAACAATTGGAGGGCAAGTCTGGTGCCAGCAGCCGCGGTAATTCCAGCTCCAATAGCGTATATTAAAGTTGTTGTGGTTAAAAAGCTCGTAGTTGAACCTTGGGCCTGGCTGGCCGGTCCGCCTCACCGCGTGTACTGGTCCGGCCGGGCCTTTCCCTCTGTGGAACCCCATGCCCTTCACTGGGCGTGGCGGGGAAACAGGACTTTTACTTTGAAAAAATTAGAGTGCTCCAGGCAGGCCTATGCTCGAATACATTAGCATGGAATAATGAAATAGGACGTGCGGTTCTATTTTGTTGGTTTCTAGGACCGCCGTAATGATTAATAGGGACAGTCGGGGGCATCAGTATTCAATTGTCAGAGGTGAAATTCTTGGATTTATTGAAGACTAACTACTGCGAAAGCATTTGCCAAGGATGTTTTCATTAATCAGGAACGAAAGTTAGGGGATCGAAGACGATCAGATACCGTCGTAGTCTTAACCATAAACTATGCCGA

>*Parametarhizium changbaiense* SGSF125 ITS

CCCCTCCTCTTACCGGGAGGGGTGAGCCGGACAACACGGCCCCCGCCGGAGGACCCAAAC

TCTTGAATTTTCGCGACTGGCTCGCGGCAGTTTGTATTCTGATTAATTCAAAACAAAATGAATCAAAACTTTCAACAACGGATCTCTTGGTTCTGGCATCGATGAAGAACGCAGCGAAATGCGATAAGTAATGTGAATTGCAGAATTCAGTGAATCATCGAATCTTTGAACGCACATTGCGCCCGCCAGTATTCTGGCGGGCATGCCTGTTCGAGCGTCATTTCAACCCTCAAGCACTGCTTGGTGTTGGGGGCCGGCGAGGTCGTCTTTCTCGACGCGCCGCCCCCGAAATGACTTGGCGGCCCCGTCGCGGCCCTTCCTCTTGCGTAGTAGCACACACCTCGCATCGGGAGCCCGACGGCGGCCACTGCCGTAAAACACCCAACTTTCTCAGAAGTTGACCTCGGATCAGGTAGGAATA

>*Parametarhizium changbaiense* SGSF125 LSU

CGTCAGAACCGCTGCGAGCCTCCACCAGAGTTTCCTCTGGCTTCACCCTATACAGGCATA

GTTCACCTTCTTTCGGGTCCGGCCCCGTATGCTCTTACTCAAATCCATCCGAGAACATCAGGATCGGTCGATGATGCGCCGAAGCTCTCACCTGCGTTCACTTTCATTACGCGTAGGGGTTTGACACCCGAACACTCGCATACGAAGACGACTCCTTGGTCCGTGTTTCAAGACGGGTCGCTGATGACCATTACGCCAGCATCCATGCAGATGCGCGAACCTCAGTCCGCCCCAGGGTATTGTGCAACGGGCTATAACACTCCCGGAGGAGCCACATTCCCGAAGCCTTTATCCCCCAAGGCAAACTGATGCTGGCCTGGACCCGGCAAAGTGCACCAGCGAGAACGCTGGATGATTCACCGGGCCCAAGTCTGGTCATGAGCGCTTCCCTTTCAACAATTTCACGTACTTTTTAACCCTCTTTTCAAAGTGCTTTTCATCTTTCGATCACTCTACTTGTGCGCTATCGGTCTCTGGCCAATATTTAGCTTTAGAAGACATATACCTCCCATTTAGAGCAGCATTCCCAAACTACTCGACTCGTCGAAGGAGCTTTACAGAGGCTCGGCATCCAACCAGACGGGGCTCTCACCCTCTATGGCGTCCCGTTCCAGGGAACTCGGAAGGCGCCTCGCCAAAAGCATCCTCTGCAAATTACAACTCGGGCACCAGGTGCCAGA

>*Parametarhizium changbaiense* SGSF125 TUB

TTTCTGGCGAGCACGGTCTCGACAGCAATGGTGTCTACAATGGTACCTCTGAGCTCCAGCTGGAGCGTATGAGCGTGTACTTTAACGAGGTGAGTGATTGAACAAGCTCATTCCAAGCCAGCTATTCTCTGGCTGCATGATCGGAGCAGCCGTAGCTAAAACATCGCTAGGCCTCTGGAAACAAATATGTTCCTCGCGCTGTTCTCGTCGATCTCGAGCCGGGCACCATGGATGCCGTTCGTGCTGGTCCTTTTGGTCAGCTTTTCCGCCCTGACAACTTTGTTTTTGGCCAGTCCGGTGCTGG

> *Parametarhizium changbaiense* SGSF125 TEF

AGTTCGAGGCTGGTATCTCCAAGGATGGCCAGACTCGTGAGCACGCTCTGCTCGCCTACA

CCCTGGGTGTCAAGCAGCTCATCGTTGCCATCAACAAGATGGACACCACCAAGTGGTCTC

AGGCCCGTTACGAGGAAATCATCAAGGAGACTTCCAACTTCATCAAGAAGGTCGGCTACA

ACCCCAAGACTGTTGCCTTCGTCCCCATCTCTGGTTTCCACGGCGACAACATGTTGCAGGCCTCCACCAACTGCCCCTGGTACAAGGGCTGGGAGAAGGAGACCAAGGCCGGCAAGTCCA

CTGGCAAGACCCTTCTCGAGGCCATTGACTCCATTGAGCCCCCCAAGCGTCCCACAGACA

AGCCCCTCCGTCTTCCCCTCCAGGATGTGTACAAGATTGGCGGTATTGGAACTGTCCCTGTCGGCCGTATCGAGACTGGTATCATCAAGCCCGGTATGGTCGTTACCTTCGCTCCTTCCAACGTCACCACTGAAGTCAAGTCCGTTGAGATGCACCACGAGCAGCTCACCGAGGGTGTCCCCGGTGACAACGTTGGCTTCAACGTGAAGAACGTTTCCGTCAAGGAAATCCGCCGTGGCAACGTCGCTGGTGACTCCAAGAACGACCCCCCCATGGGTGCCGCTTCTTTTGATGCCCAGGTCATTGTCCTCAACCACCCCGGCCAGGTCGGTGCTGGTTACGCTCCCGTCCTCGATTGCCACACTGCCCACATTGCTTGCAAGTTCGCCGAGATCAAGGAGAAGATTGACCGACGTACCGGTAAGGCTGTCGAGGCTGCCCCCAAGTTCATCAAGTCTGGAGACTCCGCCATCGTCAAGATGG

*>Parametarhizium changbaiense* SGSF125 RPB1

AGATTGTCTGCCATAACTGTAGCAAAGTGTTGGCTGATACTGTTGGTCTATCCCAACTCTCCATAGGCTTTTTTGTAGGCTTGACTGATTGCTAACACTCTTTTTTCTTTTTTTTTCTCAGAGCGATCCCGAGTTTGTCACCGCCATCGGTACTCGAGACGCTAAGCTGCGCTTCAATCGTGTCTGGGCAGTCTGCAAGAAGAAGCGGAGATGTGAAAACGAGGACCGCACCGAGAAGAACGAAGAGGAATTCGCCCCTGGTATGAAGCCTGTCGCGCACAACCACGGTGGCTGCGGCAACGTGCAACCCCAAGTACGACAGGCGGCTCTCCAACTGAAGGCTGCTTTCGATGTTGCTCAAGAGGATGGCCCCAAGAGGCGAGAGACGGTGCCCATCACCCCGGAAATGGCTCACGGCATTTTGAAGAGAATATCCGAGGAAGACTTGCGACATATGGGACTCAACTCGGACTATGCGCGTCCCGAGTGGATGATCCTCACTGTTCTGCCTGTTCCCCCTCCCCCCGTGCGTCCTAGTATCTCCATGGATGGTACCGGCACCGGCATGCGAAACGAAGACGATCTGACATACAAGCTTGGTGATATTATCCGTGCCAATGGTAACGTGAAGCAGGCTATCCGTGAAGGATCTCCTCAGCACATTGCCAGAGATTTTGAAGAGTTGCTTCAGTACCACGTGGCAACGTACATGGACAACGATATTGCTGGCCAGCCGCGTGCCCTCCAAAAGAGTGGCCGTCCCGTCAAGGCCATCAGAGCTCGATTAAAGGGCAAGGAGGGTCGATTGAGAGGTAACTTGATGGGAAAGCGAGTGGATTTCTCGGCTCGTACCGTCATCACTGGTGATGCCAATCTTTCTCTTCACGAAGTCGGTGTTCCTCGCAGCATCGCTAGAACGCTCACATATCCGGAGACAGTCACGCCTTACAACATCGGCAAGCTGCATCAGCTTGTGGAGAACGGGCCAAACGAGCATCCAGGTGCCAAGTACGTCATTCGCTCTGACGGCACTCGCATTGATTTGCGACATCACCGACGAGCTGC

>*Parametarhizium changbaiense* SGSF125 RPB2

GGGCTTACCTGGTTATGGTCAGGGAATGGAATAATGCTAGCGCAAATGCCGAGAATCATG

CTAGGGTGGATTTCGCAGTGAGTGTACATGTGTGTCGTAGGGTTGGTCTTTGTCTTGAGA

CGTCTGTTTAGATCATCACCAAGATCATCGTCTAGCGCAACGCCAGCTTTCTGCAGACGG

TATAACTCAAGGTCTTCAGGCGTCATACAGATCATAGATGTCTCTTCTTCCTCGGCATCTAGATACTCAACCGCACCTGCCCGAATCAAGCCTTCCCAACCAATCCTTTCGTCAGGGTCCTCTGGGGGTTCAGCTTGCTCCTTGGCCAGCTTGTTGACTAGCTCTTTTGATAAGACAAGGTGTCCCTTTTCGATGCCCGTCTCCGGGTCATCTTCTTGCTGGACCGTGAAGACTGGTCGCATGACTCGGCCAGCATCAGAGAAGATCTTAAACTCTTGGTCCCTAATTTCTCGTACCAGTGAAACCTCATACTGAAGGTAAGACTTTCGTCGAGTGTCCAAAACTTGGCTGACAAGATGTTTCGGGTCCTGATGCACACCCACCCATACACCATTGACAAAGATCTTTGTTGCATGGGGATATCTCAGCGGCTCATACTCCTCCACGACTTCCATACCTCGGTTGATCATGAACTCAATCAAGGGCTCCGAAGGCGAACCCACACTGACGTAACACATCAAAGACAAATTCTTTACCAGACCACAAGCTTGGCCTTCGGGTGTTTCAGCAGGACAGACAAGGCCCCAATGTGTATTATGCAGTTGTCGCGGCTTGGCAAGCTTACCATCTCTGCCGATGGGGGTATTGGTACGTCGCAAGTGAGAAAGCGTCGAGGCAAAAGTATAACGATTCAATACTTGTGACACGCCAGCCGTCGAGCTCATAGCCTTCTTCTGGTCACCCCAGTTACCAGTGGCGAGAGAATATTTCAAGCCGTTCGAGAGAGTTCCAGGCTTGATACCGACTGCCAAGTTGAAGTGTCTGTTGCCTTCAACACATCGGCGCAAGTAATTGGATAACTCTGTATTCATTC

>*Parametarhizium hingganense* SGSF355 SSU

TCGGCATAGTTTATGGTTAAGACTACGACGGTATCTGATCGTCTTCGATCCCCTAACTTTCGTTCCTGATTAATGAAAACATCCTTGGCAAATGCTTTCGCAGTAGTTAGTCTTCAATAAATCCAGAATTTCACCTCTGACAATTGAATACTGATGCCCCCGACTGTCCCTATTAATCATTACGGCGGTCCTAGAAACCAACAAAATAGAACCGCACGTCCTATTTCATTATTCCATGCTAATGTATTCGAGCATAGGCCTGCCTGGAGCACTCTAATTTTTTCAAAGTAAAAGTCCTGTTTCCCCGCCACGCCCAGTGAAGGGCATGGGGTTCCACAGAGGGAAAGGCCCGGCCGGACCAGTACACGCGTGAGGCGGACCGGCCAGCCAGGCCCAAGGTTCAACTACGAGCTTTTTAACCACAACAACTTTAATATACGCTATTGGAGCTGGAATTACCGCGGCTGCTGGCACCAGACTTGCCCTCCAATTGTTCCTCGTTAAGGGATTTAAATTGTACTCATTCCAATTACAAGACCCAAAAGAGCCCTGTATCAGTATTTATTGTCACTACCTCCCCGAATCGGGATTGGGTAATTTGCGCGCCTGCTGCCTTCCTTGGATGTAGTAGCCGTTTCTCAGGCTCCTTCTCCGGGGTCGAGCCCTAACCCTCCGTTACCCGTTGTAACCATGTTTGGCCACTACCCAAACATCGAAAGTTGATAGGGAAGAAATTTGAATGAACCATCGCCGGCGCAAGGCCATGCGATTCGAGAAGTTATCATGAGTCACCAAGGAGCCCAGAGGGCATTGGTTTTTAATCTAATAAATACATCCCTTCCGAAGTCGGGATTTGTAGCATGTATTAGCTCTAGAATTACCACGGTTATCCAAGTAGTAATATACTATCAAA

>*Parametarhizium hingganense* SGSF355 ITS

CGTTGCTTCGGCGGGTCTCGGCCCCGGCGCTGCCGCTCCCCTTGCCGGGAGGGGTAAGCC

GGACAACACGGCCCCCGCCGGAGGACCCAAACTCTTGAATTTTTCCGCGGACTGGCTTCG

CGGCAGTTTGTATTCTGATTACTTCAAAACAAAAATGAATCAAAACTTTCAACAACGGATCTCTTGGTTCTGGCATCGATGAAGAACGCAGCGAAATGCGATAAGTAATGTGAATTGCAGAATTCAGTGAATCATCGAATCTTTGAACGCACATTGCGCCCGCCAGTATTCTGGCGGGCATGCCTGTTCGAGCGTCATTTCAACCCTCAAGCACTGCTTGGTGTTGGGGACCGGCGAGGTCGTCTCTCTCGACGCGCCGCCCCCGAAATGACTTGGCGGCCCCGTCGCGGCCCTTCCTCTTGCGTAGTAGCACACACCTCGCATCGGGAGCCCGACGGCGGCCACTGCCGT

>*Parametarhizium hingganense* SGSF355 LSU

CGTATGCTCTTACTCAAATCCATCCGAGAACATCAGGATCGGTCGATGATGCGCCGAAGCTCTCACCTGCGTTCACTTTCATTACGCGTAGGGGTTTGACACCCGAACACTCGCATACGAAGACGACTCCTTGGTCCGTGTTTCAAGACGGGTCGCTGATGACCATTACGCCAGCATCCATGCAGATGCGCGAACCTCAGTCCGCCCCAGGATATTATGCAACGGGCTATAACACTCCCGGAGGAGCCACATTCCCGAAGCCTTTATCCCCCGGGGCAAACTGATGCTGGCCTGGACCCGGCAAAGTGCACCAGCGAGAACGCTGGATGATTCACCGGGCCCAAGTCTGGTCATGAGCGCTTCCCTTTCAACAATTTCACGTACTTTTTAACCCTCTTTTCAAAGTGCTTTTCATCTTTCGATCACTCTACTTGTGCGCTATCGGTCTCTGGCCAATATTTAGCTTTAGAAGACATATACCTCCCATTTAGAGCAGCATTCCCAAACTACTCGACTCGTCGAAGGAGCTTTACAGAGGCTCGGCATCCAACCAGACGGGGCTCTCACCCTCTATGGCGTCCCGTTCCAGGGAACTCGGAAGGCGCCTCGCCAAAAGCATCCTCTGCAAATTACAACTCGGGCACCAGGTGCCAGATT

>*Parametarhizium hingganense* SGSF355 TUB

TGTTGAGCCCCTGATATTGGTACCCCGCCGGCTATGTCGCGTTCAGCGCCCAACGGGCCAGACGAGGCAAAACGCGATGCAGGTTCACCTCCAGACCGGCCAGTGCGTAAGTGGGAAACTCACACGTAGGGTAACCAAATGGTGCTGCTTTCTGGCAGACCATTTCTGGCGAGCACGGTCTTGACAGCAATGGTGTCTACAATGGTACCTCTGAGCTTCAGCTGGAGCGTATGAGCGTGTACTTTAACGAGGTGAG

>*Parametarhizium hingganense* SGSF355 TEF

CGCCTACACCCTGGGTGTCAAGCAGCTCATTGTTGCCATCAACAAGATGGACACCACCAA

GTGGTCTCAGGCCCGTTACGAGGAAATCATCAAGGAGACTTCCAACTTCATCAAGAAGGT

CGGCTACAACCCCAAGACTGTTGCCTTCGTCCCCATCTCTGGTTTCCACGGCGACAACATGTTGCAGGCTTCCACCAACTGCCCCTGGTACAAGGGTTGGGAGAAGGAGACCAAGGCCGG

CAAGGCCACTGGCAAGACCCTTCTCGAGGCCATTGACTCCATTGAGGCCCCCAAGCGTCC

CACAGACAAGCCCCTCCGTCTTCCCCTCCAGGATGTGTACAAGATTGGCGGTATCGGAACTGTCCCTGTCGGCCGTATCGAGACTGGTGTCCTCCGGCCCAATATGGTCATTACCTTCGCTCCTTCCAACGTCACCACTGAATGCAAGTCCGTTGAGATGCACCACGAGCAGCTCTCTGAGGGTGTTCCCGGTGACAACGTCGGCTTCAACGTGAAGAACGTTTCTGTCAAGGAAATCCGCCGTGGCAACGTCGCTGGTGACGCCAAGGACAAACCCCCCCAGGGTGCCGCTTCTTTCGATGCCAGGTCATTGTCCTCAACCACCCCGGCCAGGTCGGTGCTGGTTACGCTCCCGTCCTCGATTGCCACACCGCCCACATTGCTTGCAAGTTCGCCGAGATCAAGGAGAAGATTGACCGACGTACCGGTAAGGCTGTCGAGTCTAACCCCAAGTTCATCAAGTCTGGAGACGCCG

>*Parametarhizium hingganense* SGSF355 RPB1

TTTTGGAGATTGTCTGCCATAACTGTAGCAAAGTGTTGGCCGATACTGTTGGTCTATCCCAACTCTCCATTAGGCTTTTTGTATGGCTTGACTGATTGCTAACACTCTTTTGTTTTTTTCCTTCAGAGCGATCCCGAGTTTGTCACCGCCATTCACACTCGAGACGCTAAGCTGCGCTTCAATCGTGTCTGGGCAGTCTGCAAGAAGAAGCGAAGATGTGAAAATGAGGACCGCACCGAGAAGAACGAAGAGGAATTCGCTCCTGGTATGAAGCCTGTCGCGCACAACCACGGTGGCTGCGGCAACGTGCAACCCCAAGTACGACAGGCGGCTCTCCAACTGAAGGCTGCCTTTGATGTTGCCCAAGAGGATGGTCCCAAGAGGCGAGAGACGGTTCCCATCACCCCGGAAATGGCGCATGGCATTCTGAAGAGAATCTCCGAGGAAGATTTGCGACACATGGGTCTCAACTCGGACTATGCACGTCCTGAATGGATGATCCTCACTGTCCTGCCTGTTCCCCCTCCTCCCGTCCGTCCTAGTATCTCCATGGATGGCACCGGCACTGGCATGCGAAACGAAGACGATTTGACATACAAGCTTGGTGACATCATCCGAGCCAATGGTAACGTGAAGCAGGCTATACGCGAAGGATCCCCTCAGCACATTGCTAGAGATTTTGAAGAGTTGCTTCAGTACCATGTGGCAACGTACATGGATAATGACATCGCTGGTCAGCCCCGTGCTCTGCAGAAGAGTGGCCGTCCCGTCAAAGCCATTCGAGCTCGATTAAAGGGCAAGGAGGGTCGATTGAGAGGTAACTTGATGGGGAAGCGTGTGGATTTCTCGGCTCGTACCGTCATCACTGGTGATGCAAATCTTTCTCTTCACGAAGTCGGTGTTCCTCGCAGCATCGCCAGGACGCTCACATACCCGGAGACAGTCACGCCTTACAACATTGGCAAGCTCCATCAGCTCGTGGAGAACGGGCCAAATGAGCACCCAGGTGCCAAGTACGTCATTCGCTCTGACGGCACGCGCATCGACTTGCGACATCACCGACGAGCCGCACAGATTTCTCTCGAATACGGCTGGAAAGTCG

>*Parametarhizium hingganense* SGSF355 RPB2

CAATTACTTGCGCCGATGTGTTGAAGGCAACCGACACTTCAACTTGGCAGTTGGTATCAAGCCTGGAACTCTCTCGAATGGCTTGAAATACTCTCTCGCCACTGGTAATTGGGGTGACCAGAAGAAGGCCATGAGCTCGACAGCTGGTGTGTCACAAGTGTTGAATCGCTATACTTTTGCCTCGACGCTTTCTCACTTGCGACGTACCAATACACCCATAGGCAGGGACGGCAAGCTTGCCAAGCCGCGACAACTGCATAACACACATTGGGGCCTTGTCTGTCCTGCTGAAACACCCGAAGGCCAAGCTTGTGGTCTGGTAAAGAACTTGTCTTTGATGTGTTACGTCAGTGTGGGCTCGCCCTCGGAACCTTTGATTGAGTTCATGATCAACCGAGGTATGGAAGTCGTGGAGGAGTATGAACCGCTGAGATATCCCCACGCGACAAAGATCTTTGTCAATGGTGTGTGGGTAGGTGTACATCAGGACCCGAAGCATCTTGTCAGCCAAGTTTTAGACACTCGACGAAAGTCCTACCTTCAGTACGAGGTGTCACTGGTCCGAGAAATCAGAGACCAGGAGTTTAAGATCTTCTCTGATGCTGGTCGAGTCATGCGACCAGTCTTCACGGTCCAGCAAGAAGATGACCCTGAGACAGGCATTGAAAAAGGCCACCTTGTCTTATCAAAAGAGCTAGTCAACAAGCTGGCCAAGGAGCAAGCTGAACCCCCAGAGGATCCTGACGAAAGGATTGGATGGGAAGGCTTGATTCGGGCAGGTGCGGTTGAGTATCTGGATGCCGAAGAAGAAGAGACATCCATGATCTGTATGACACCTGAAGACCTCGAGCTATACCGCCTGCAGAAAGCTGGCGTTGCGCTAGACGATGATCTTGGCGATGATCTGAACAGACGTCTCAAGACAAAGACGAACCCCACGACACACATGTACACTCACTGCGAAATTCACCCCAGCATGATTCTCGGCATTTGCGCCAGCATTATT
